# Absence of dehydration due to superionic transition at Earth’s core-mantle boundary

**DOI:** 10.1126/sciadv.aeb3006

**Published:** 2026-01-28

**Authors:** Yu He, Wei Zhang, Qingyang Hu, Shichuan Sun, Jiaqi Hu, Daohong Liu, Li Zhou, Lidong Dai, Duck Young Kim, Simon A. T. Redfern, Yun Liu, Heping Li, Ho-kwang Mao

**Affiliations:** ^1^ State Key Laboratory of Critical Mineral Research and Exploration, Institute of Geochemistry, Chinese Academy of Sciences, Guiyang 550081, China.; ^2^ Key Laboratory of High-Temperature and High-Pressure Study of the Earth’s Interior, Institute of Geochemistry, Chinese Academy of Sciences, Guiyang 550081, Guizhou, China.; ^3^ Center for High Pressure Science and Technology Advanced Research, Shanghai 201203, China.; ^4^ School of Geography and Environmental Science (School of Karst Science), Guizhou Normal University, Guiyang 550025, China.; ^5^ Asian School of the Environment, Nanyang Technological University, 50 Nanyang Avenue, Singapore 639798, Singapore.; ^6^ University of Chinese Academy of Sciences, Beijing 100049, China.; ^7^ International Center for Planetary Science, College of Earth Sciences, Chengdu University of Technology, Chengdu 610059, China.

## Abstract

The properties and stability of hydrous phases are crucial to unraveling the mysteries of the deep water cycle. Under deep lower mantle conditions, water and hydrous phases transition into a superionic state. However, superionic effect on their stability and dehydration behavior remains unclear. Using ab initio and deep learning potential molecular dynamics simulations, we discovered a doubly superionic transition in δ-AlOOH, characterized by the highly diffusive behavior of both hydrogen and aluminum ions within the oxygen sublattice. These highly diffusive elements contribute external entropy to the system, stabilizing the structure at 140 GPa and 3800 K. Our free-energy calculations reveal that water tends to freeze under deep lower mantle conditions, so dehydration becomes energetically and kinetically unfavorable even under core-mantle boundary (CMB) conditions. This implies that superionic water may accumulate in the deep lower mantle over geologic time, forming a long-term reservoir at the base of the mantle.

## Teaser

Dehydration becomes unfavorable due to superionic transition, resulting in water reservoir in the deep lower mantle.

## INTRODUCTION

Water, in the form of hydrous minerals such as chlorite, amphibole, and serpentine, enters the mantle by subducting oceanic plates. During this process, most hydrous minerals dehydrate at high temperatures, releasing fluids which contribute to volcanism in island arcs (*1*–*3*). The released water reacts with iron-rich olivine to produce natural hydrogen (H_2_), which is increasingly recognized as an important renewable clean energy resource (*4*, *5*). Some hydrous minerals, however, transform to high-density hydrous phases in cold subduction slabs such as phase H (MgSiO_4_H_2_) (*6*–*7*), δ-AlOOH (*8*–*11*), pyrite-type (py) FeO_2_H*_x_* (*x* ≤ 1) (*12*–*15*), and hydrous SiO_2_ (*16*–*20*). In these hydrous phases, asymmetric O─H covalent bonds (hydroxyl) gradually transition to symmetric O─H─O ionic bonds as pressure increases (*21*–*23*). These hydrous phases present enhanced stability and can coexist with bridgmanite and davemaoite (*9*, *10*, *20*), enabling water to be transported into the lowermost mantle.

Water (*24*–*28*) and hydrous phases (py-FeO_2_H*_x_*, δ-AlOOH, and hydrous aluminous SiO_2_) (*29*–*32*) undergo a transition to a superionic (SI) state under lower mantle conditions. In a SI state, liquid-like proton diffusion within the crystal lattice, leading to high electrical conductivities (*30*). Furthermore, the presence of highly diffusive protons causes elastic softening and reduces shear wave velocities (*33*). In SI H_2_O, the additional entropy contributed by highly diffusive protons induces structural changes and increases the liquid’s temperature (*24*–*28*). However, the SI effect on thermal stability and dehydration process of hydrous phase is still unknown.

The stability and dehydration of hydrous phases are also critical for the generation and transportation of H_2_ in deep lower mantle. Dehydrogenation has been observed during phase transition from ε-FeOOH (CaCl_2_ structure) to py-FeO_2_H*_x_* (*x* ≤ 1) at ~70 GPa (*14*, *15*, *34*, *35*). In addition, released water reacts with iron and iron oxides to produce py-FeO_2_H*_x_* (*14*, *36*, *37*), presenting notably high density and low seismic velocities, comparable to the properties of ultralow velocities zone above CMB (*15*). These reactions can also produce iron hydride, which may transport hydrogen into Earth’s outer core (*15*, *37*, *38*). It is interesting that hydrogen from deep mantle is found to be associated with high ^3^He content, suggesting primordial resource (*4*, *39*, *40*). Alternatively, primordial hydrogen may be released from Earth’s core through core-mantle reactions (*4*). However, the relationship between subducted water and deep-released hydrogen is not established.

## RESULTS

### State of H_2_O in the lower mantle

**Fig. 1.**
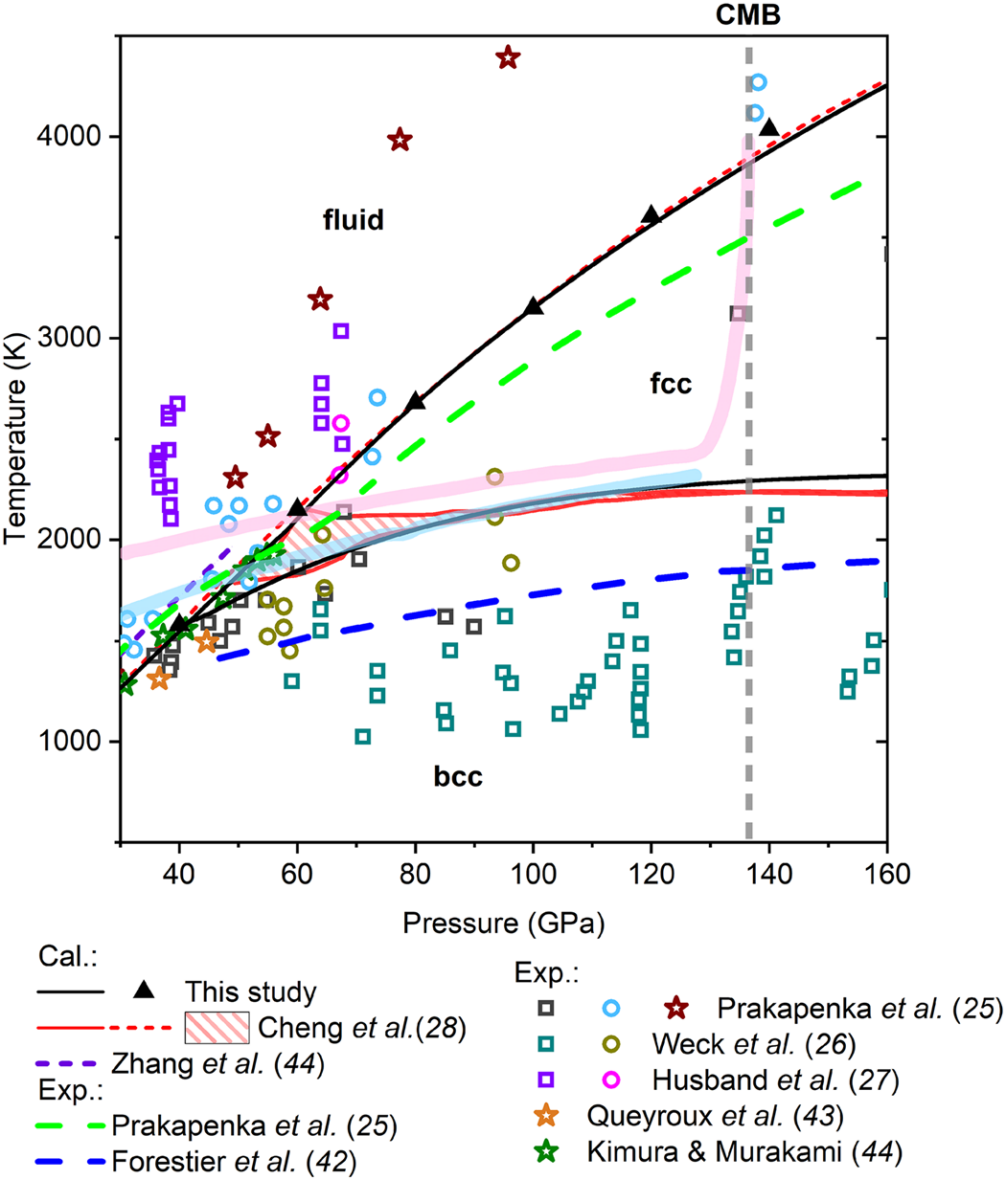
Calculated phase diagram of H_2_O at 30 to 160 GPa and 1000 to 4500 K. Calculated phase boundaries for bcc-fcc and fcc-fluid transitions are shown with black curves. The results of solid-liquid coexistence simulations are shown with black triangles. The data are compared with previous experimental (*25*–*27*, *42*, *43*) and computational (*28*, *44*) studies. The empty squares, circles, and stars present the observation of bcc, fcc, and fluid H_2_O, respectively (*25*–*27*, *41*–*44*). Experimental predicted bcc-fcc phase boundaries are shown with blue (*42*) and green (*25*) dashed curves. The red (*28*) and purple (*81*) short dashed curves present the calculated melting curves. The solid red curve is the calculated bcc-fcc phase boundary, with uncertainty region shown with red hatched area (*28*). Normal mantle and subducting slab geotherms are shown with light pink (*82*) and light blue (*83*) curves, respectively.

The phase diagram of H_2_O ice is important for the dehydration process in Earth’s interior. Thus, we calculated Gibbs free energies of body-centered cubic (bcc; ice X, bcc SI), face-centered cubic (fcc; ice XVIII), and liquid water at pressures ranging from 30 to 160 GPa and temperatures between 1000 and 4500 K by conducting deep potential molecular dynamics (DPMD) simulations. The Gibbs free energies of SI phases were calculated via a two-step thermodynamic integration (TI) method, which accounts for both solid and liquid features of a SI phase (*41*). On the basis of the computed free energy differences between phases, we constrained the phase boundaries of H_2_O, as illustrated in Fig. 1 (solid curves). Below ~42 GPa, bcc phase transforms directly to the liquid at temperature above ~1600 K. In the pressures between ~42 and 160 GPa, the fcc phase becomes more stable than the bcc phase at temperature exceeding ~1600 to 2300 K. Notably, the fcc phase exhibits a wide stability field, with a melting temperature surpassing 4200 K at 160 GPa. In addition, consistent freezing points of liquid H_2_O are provided using two-phase coexisting method (Fig. 1 and fig. S1).

Our calculated phase boundaries are consistent with previous theoretical predictions for dense H_2_O by comparing chemical potentials of SI phases with liquid water in coexisting systems (*28*). However, experimental studies report differing stability fields for the bcc and fcc phases (Fig. 1). Two independent laser-heated diamond anvil cell (DAC) experiments, using different heat absorbers, show substantial discrepancies in the bcc-fcc transition temperatures (*25*, *26*, *42*). These variations likely arise from temperature gradients during laser heating (*42*). Our predicted phase boundary lies between these two experimental results but aligns more closely with the result of lower transition temperatures (*26*, *42*). In addition, our calculated melting curve agrees well with CO_2_ laser–heated DAC experiments (*43*, *44*), where H_2_O samples were directly heated, avoiding potential reactions between water and the absorber.

Given the extensive stability field of SI fcc H_2_O, liquid water may undergo a liquid-to-SI phase transition in Earth’s deep lower mantle. Compared to normal mantle and subducting slab geotherms, liquid water freezes at depths above ~1240 and ~1450 km, making an icy world in deep lower mantle. At CMB conditions, the melting temperature of fcc H_2_O reaches ~3870 K. Throughout most of the deep lower mantle, H_2_O exists in a SI state, which could substantially influence the dehydration processes of hydrous phases. Therefore, we investigated the stability of δ-AlOOH and calculated the Gibbs free energy of its dehydration reaction with SI H_2_O as reference phases.

### Double SI transition in δ-AlOOH

δ-AlOOH, with CaCl_2_-type structure, is stable under deep lower mantle conditions (*8*–*11*). As an end member, it forms CaCl_2_-type AlOOH-Mg_0.5_Si_0.5_OOH-FeOOH and AlOOH-SiO_2_ solid solution phases (*6*, *9*, *18*, *20*, *45*). In addition, the incorporation of Al markedly increases the water solubility in stishovite and post-stishovite, making hydrous aluminous SiO_2_ one of the dominant water hosts in the lower mantle (*18*, *20*). Therefore, understanding the Al-O-H interaction is key to elucidating the properties of hydrous phases in the lower mantle.

**Fig. 2.**
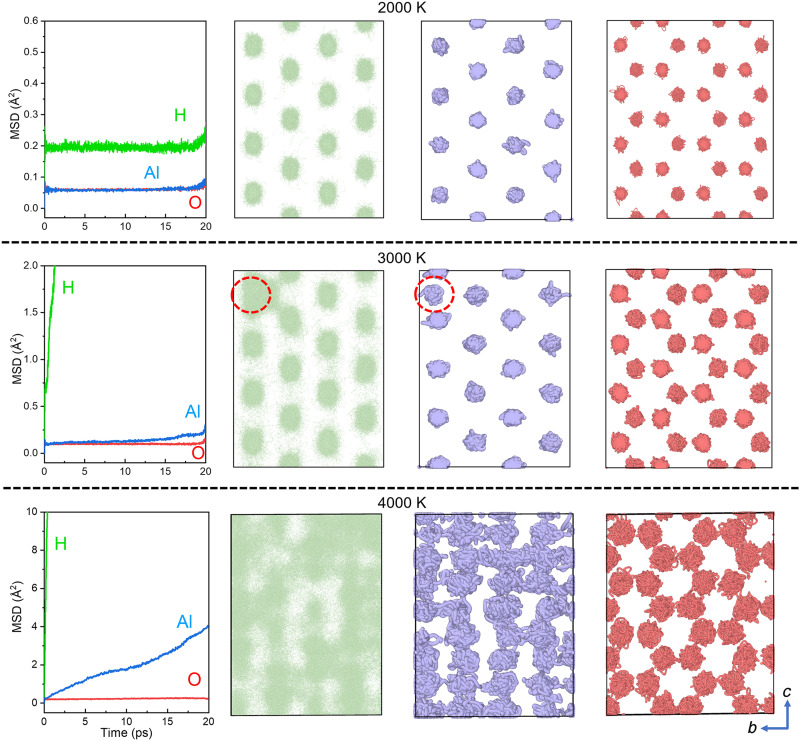
The MSDs and trajectories of H, Al, and O ions in δ-AlOOH upon double SI transition with increasing temperature from 2000 to 4000 K at 130 GPa. The MSDs of H, Al, and O ions are exhibited in the left side with green, blue, and red curves. The linear increase of MSDs with simulation time at 3000 K for H^+^ and 4000 K for Al^3+^ indicates fast ionic diffusion. The trajectories of H^+^, Al^3+^, and O^2−^ are shown with green, blue, and red spheres on the right side. The disordered distributions of H^+^ and Al^3+^ are observed above 3000 K indicating a SI transition. The migration of Al^3+^ is also observed at 3000 K noted with red dashed circles resulting in enhanced proton diffusion along the *b* axis.

Here, we conducted ab initio molecular dynamics (AIMD) simulations on δ-AlOOH at 130 GPa and 2000 to 4000 K. Consistent with previous studies (*32*), we observed a SI transition. Protons become highly diffusive at 3000 K (Fig. 2). In addition, we detected Al^3+^ migration along the *b* axis, which promotes proton diffusion in the same direction. This suggests that proton diffusion provides external migration sites for Al^3+^ (fig. S2). At 4000 K, the diffusion of Al^3+^ is notable with mean squared displacement (MSD) increasing linearly with simulation time. Both H^+^ and Al^3+^ exhibit liquid-like diffusion within the oxygen sublattice, indicating a doubly SI transition in δ-AlOOH (Fig. 2). The diffusion of Al^3+^ reduces the anisotropy of H^+^ diffusion, exhibited by the H^+^ MSDs along different lattice directions (fig. S3).

**Fig. 3.**
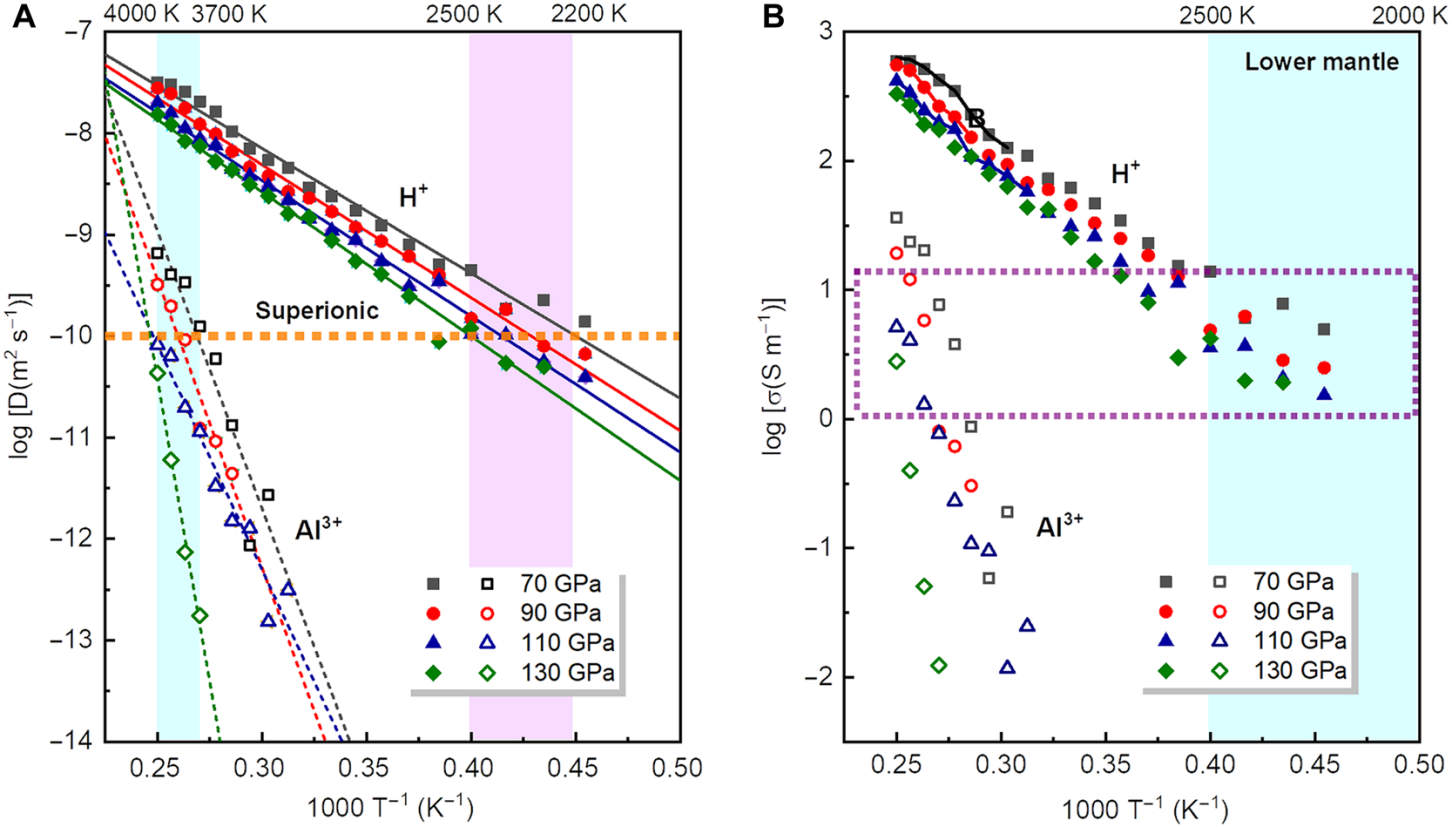
Diffusion coefficients and ionic conductivities of δ-AlOOH at 70 to 130 GPa and 2000 to 4000 K. The calculated (DPMD) diffusion coefficients (**A**) and ionic conductivities (**B**) of H^+^ and Al^3+^ are shown with filled and empty symbols, respectively. The diffusion coefficients are fitted with the Arrhenius equation (solid and dashed lines). The dashed orange line represents the typical diffusion coefficient for SI materials. On the basis of this line, the predicted SI transition temperatures are shown with pink and cyan region. The total ionic conductivities of δ-AlOOH are shown with solid curves suggesting the contribution of Al^3+^ is insignificant. The conductivities of δ-AlOOH under lower mantle conditions (cyan region) are enclosed in dashed purple rectangle.

Large-scale molecular dynamic simulations based on deep learning potentials (DPs) were used to investigate diffusion of H^+^ and Al^3+^ in δ-AlOOH at ~70 to 130 GPa and ~1500 to 4000 K. The calculated densities are consistent with AIMD results (fig. S4). Diffusion coefficients were calculated using MSDs (fig. S5) and fitted with the Arrhenius equation (Fig. 3A). On the basis of typical ionic diffusion coefficients (~10^−11^ to 10^−10^ m^2^ s^−1^) in SI materials (*46*), we estimated the SI transition temperatures. The transition temperature for fast H^+^ diffusion increases from ~2050 to ~2270 K with rising pressure from 70 to 130 GPa, whereas Al^3+^ diffusion becomes notable only at very high temperatures above ~3570 K. Using the diffusion coefficients, we calculated the ionic conductivities of δ-AlOOH (Fig. 3B). Although the contribution of Al^3+^ to the total conductivity is marginal, the ionic conductivity of δ-AlOOH reaches ~600 S m^−1^ before melting. Under lower mantle conditions, the conductivities range from 1 to 10 S m^−1^.

### The stability of δ-AlOOH

**Fig. 4.**
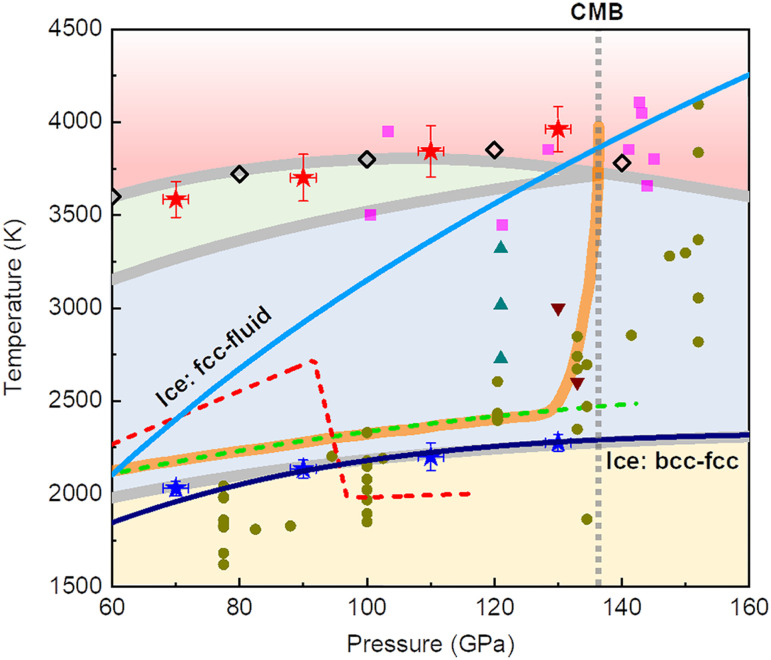
Phase diagram of δ-AlOOH in comparison with H_2_O at 60 to 160 GPa and 1500 to 4500 K. Phase transitions from solid (yellow) to SI-I (blue) to SI-II (green) and to liquid (red). The melting temperatures of δ-AlOOH predicted by two-phase coexistence simulations are shown with black empty diamonds. Green (*10*) and red (*11*) dashed curves represent the instability of δ-AlOOH suggested by high-pressure experiments. The stability field of hydrous SiO_2_, pyrite FeO_2_H, and hydrous aluminous SiO_2_ are shown with solid brown circles (*19*), cyan triangles (*30*), dark red inverted triangles (*15*), and magenta squares (*20*), respectively. The *P*-*V* of highly H^+^ and Al^3+^ diffusion (SI transition) is shown with open blue and red stars (with errors from the linearly fitted diffusion coefficients). Phase boundaries of ice X (bcc) to ice XVIII (fcc), and ice XVIII (fcc) to liquid are exhibited with dark blue and light blue curves. Lower mantle normal geotherm is shown with an orange belt (*82*). The geotherm increases dramatically to over 3500 to 3800 K in CMB regions.

We calculated the Gibbs free energies for solid δ-AlOOH, the SI phase with diffusive H^+^ (SI-I), the doubly SI phase with diffusive H^+^ and Al^3+^ (SI-II), and the liquid phase using a two-step TI method. The resulting phase diagram is shown in Fig. 4. Ionic diffusion within the lattice stabilizes the solid structure, resulting in a high melting temperature of approximately 3800 K under core-mantle boundary (CMB) conditions. Two-phase coexisting simulations (SI-II and liquid) also predicted consistent melting temperatures for δ-AlOOH, eliminating the superheating effect (fig. S6) (*33*). At pressures above ~140 GPa, the SI-II phase becomes unstable, and the SI-I phase transitions directly to the liquid phase at ~3900 K. It may be caused by the instability of Al-site vacancies with increasing pressure. The free energy calculation results also suggest that the predicted SI transition based on diffusion coefficients (red stars) may be an artifact of not accounting for the superheating effect. Here, we demonstrate that the two-step TI method provides accurate phase boundaries for solid-SI-liquid transitions.

As discussed above, rather than liquid H_2_O, SI H_2_O ice is more stable in the deep lower mantle. In this scenario, δ-AlOOH may dehydrate to produce SI H_2_O (fcc and bcc) and Al_2_O_3_ (Ru_2_O_3_-II structure). Therefore, we calculated the relative Gibbs free energy differences (Δ*G*) for the following reaction at pressures of 80–140 GPa and temperatures of 2000 to 3600 K.

$$\text{2AlOOH}\;\left(\delta \right)={\text{Al}}_{2}O_{3}\;\left({\text{Ru}}_{2}O_{3}-\text{II}\right)+H_{2}O\;\left(\text{fcc}\;\text{an}\;\text{bcc}\right)$$

Gibbs free energies for individual phases are calculated by nonequilibrium TI (NeTI) method, and the energy differences were calculated

$$\mathrm{\Delta G}=G_{H2O}+G_{\text{Al}2O3}-2G_{\text{AlOOH}}$$

**Fig. 5.**
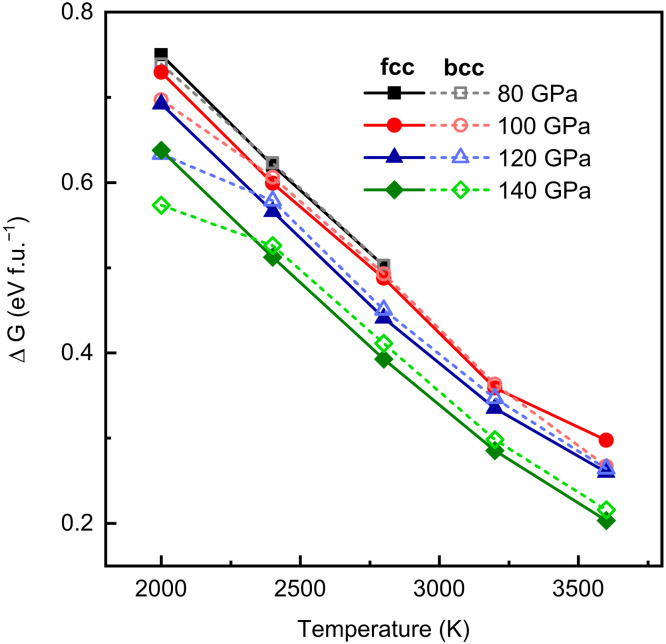
Calculated Gibbs free energy differences (Δ*G*) of the dehydration reaction of δ-AlOOH at 80 to 140 GPa and 2000 to 3600 K. Both fcc (solid symbols) and bcc H_2_O (empty symbols) are considered as the products of dehydration. f.u., formula unit.

The energy differences decrease with increasing temperature and pressure; however, they remain positive (Fig. 5), indicating that the dehydration process is energetically unfavorable.

The dehydration mechanism of hydrous minerals is well understood at ambient pressure. At elevated temperatures, H^+^ becomes diffusive and reacts with hydroxyls to form water molecules at interstitial and/or dislocation sites within the lattice. Subsequently, these water molecules are released from the lattice, accompanied by a recrystallization process (*47*, *48*). However, hydroxyl groups transform into symmetric O-H-O configurations (fig. S7) and H_2_O exists in a SI state, which prevents its release from the lattice. Our simulations also indicate that oxygen is stable at lattice sites without significant diffusion even at 4000 K (Fig. 2). As a result, dehydration under deep lower mantle conditions also becomes kinetically unfavorable.

## DISCUSSION

The stabilities of hydrous phases under deep lower mantle conditions were studied using laser heated DACs. The dehydration of δ-AlOOH occurs at temperatures approximately 2400 K under CMB conditions, based on the formation of Al_2_O_3_ (*10*, *11*). On the other hand, py-FeO_2_H*_x_* (*x* < 1) remains stable above 3000 K at 120 to 130 GPa (*15*, *30*), and aluminous SiO_2_ containing 0.9 to 2.6 wt % H_2_O coexists with melts and remains stable even at temperatures close to 4000 K under CMB conditions (*20*).

Several experimental studies report dehydration temperatures that overlap with the stability field of SI H_2_O (Fig. 4), implying that SI ice should form during dehydration and may potentially be detected with sufficient heating duration. However, none of these experiments have observed any ice phase, creating a puzzling discrepancy. This inconsistency may arise from reactions between δ-AlOOH and the surrounding pressure medium or heat absorber during experiments. In studies of δ-AlOOH dehydration, NaCl was used as both a pressure medium and thermal insulator. However, existing research indicates that NaCl can dissolve in dense H_2_O under these conditions (*49*, *50*). Consequently, H_2_O may partition between different phases at high temperatures, with proton likely diffusing into the pressure medium and accelerating interdiffusion processes.

Given the significant temperature gradients and potential chemical interactions between SI phases and pressure media in high-pressure experiments, theoretical constraints on the stability of SI hydrous phases become critically important. In this study, we demonstrate that the stability and dehydration behavior of δ-AlOOH can be reliably determined by calculating the free energy difference. Our results reveal that the state of H_2_O fundamentally controls dehydration processes in the deep lower mantle. Specifically, the lower mantle geotherm is basically below the freezing point of liquid H_2_O, thus this thermodynamic inhibition of dehydration is broadly applicable to other hydrous phases in the lower mantle.

**Fig. 6.**
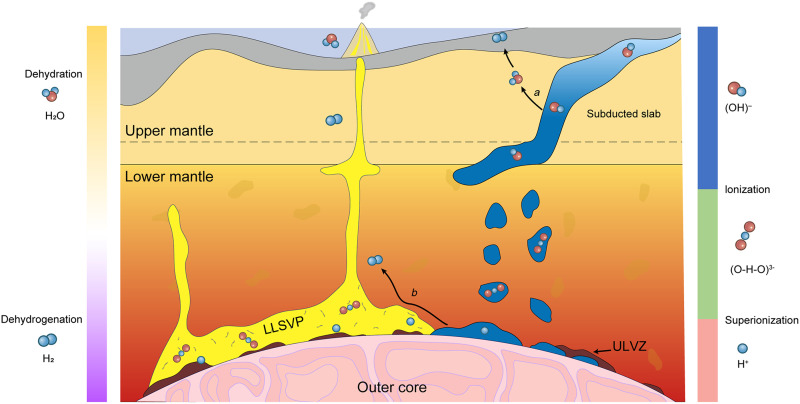
Deep water reservoir and circulation. Water can be transported into Earth’s interior by subduction slabs in the form of hydroxyls. In upper mantle, the dehydration of hydrous minerals leads to serpentinization and releases H_2_. Covalent O─H bonds undergo symmetric ionization under lower mantle conditions. In CMB regions, SI transition results in high stability of hydrous phases and makes lower mantle a potential water reservoir. Reactions between hydrous phases and iron oxides produce deep-seated H_2_, which can be transported back to Earth’s surface by mantle plume.

Dehydration and dehydrogenation processes are crucial for understanding the water and hydrogen cycles in Earth’s interior (Fig. 6). Our study demonstrates that these processes are strongly influenced by the state of water. Under upper mantle conditions, water exists in liquid form, and hydrous phases dehydrate during subduction. The released water promotes serpentinization and can oxidize ferrous ions in minerals, generating natural H_2_. In contrast, under lower mantle conditions, the symmetrization (ionization) of O─H bonds (*21*–*23*) and the SI transition (*30*–*32*) significantly enhance the stability of hydrous phases. Notably, H_2_O exists in a SI state even under CMB conditions (Fig. 4), making dehydration both energetically and kinetically unfavorable. Beyond our theoretical results, recent experimental studies also suggest the stability of hydrous silicate at CMB (*17*–*20*). Because of the high stability of hydrous phases, some ancient water may have been frozen in the deep lower mantle throughout Earth’s history.

On the other hand, experimental and computational studies suggest partial dehydrogenation from ε-FeOOH during the formation of py-FeO_2_H*_x_* (*x* < 1) at pressures exceeding ~70 GPa (*14*, *15*, *34*, *35*). Under lowermost mantle, Al^3+^ ions become highly diffusive in δ-AlOOH, promoting interdiffusion between Al^3+^ and Fe^3+^. The presence of Fe^3+^ in hydrous phases may lead to the formation of py-FeO_2_H*_x_* and gradual release of H_2_ (*41*). Alternatively, water-bearing phases are transported to the lowermost mantle, where they react with Fe from the outer core to produce H_2_, iron hydride, and iron oxides (*36*, *37*, *51*). The released H_2_ can be transported back to Earth’s surface by mantle plumes connecting large low shear velocity provinces (LLSVPs) to locations of hotspot volcanism (Fig. 6) (*52*). Hydrogen isotopic anomalies have been observed in natural H_2_ (*4*) and ocean-island basalts (OIBs) (*39*, *40*), accompanied by the enrichment of ^3^He, a primordial isotope incorporated into Earth during its accretion. The SI effect facilitates the mixing of subducted hydrogen with primordial hydrogen, which is characterized by a low deuterium-to-hydrogen (D/H) ratio. This primordial hydrogen may have been introduced into the deep Earth through an ingassing process from the atmosphere during the early stages of Earth’s formation (*53*). Coincidentally, helium (He) can also be encapsulated within the same structures (CaCl_2_ and pyrite structures) as hydrous phases under lower mantle conditions (*54*, *55*). The dehydrogenation process may also promote the leakage of He from the lattice, resulting in the enriched ^3^He signatures observed in volcanic/magmatic natural H_2_ and OIB.

LLSVPs above CMB, presenting low velocity (*56*) and sharp boundary (*57*) being distinct from surrounding mantle, are considered to be caused by thermochemical heterogeneity composed of primordial material (*58*) and/or the accumulation of subducted oceanic crust (*59*). The seismic velocity features of LLSVPs can also be explained by the presence of hydrous phases (*60*) or hydrous bridgmanite (*61*). Therefore, both geophysical and geochemical observations suggest that LLSVPs may act as sinks for ancient water in the lower mantle, with the dehydrogenation process leading to the release of H_2_ and He with primordial signatures. The ancient water reservoir located deep within Earth may be crucial to reveal the mysteries of water and hydrogen deep cycling from the early stages of Earth’s history to the present.

## MATERIALS AND METHODS

### AIMD simulations on SI transition in δ-AlOOH

AIMD simulations are widely used to investigate SI transitions in Earth and planetary interiors. This study employs AIMD simulations based on density functional theory (DFT) (*62*) using the Vienna Ab Initio Simulation Package (*62*). We used atomic potentials generated using the projector augmented-wave method (*63*, *64*) within the generalized gradient approximations (*65*). The energy cutoff was set to 600 eV and Brillouin zone sampling was performed at the Γ point. A 2 × 2 × 4 supercell (128 atoms) of δ-AlOOH with a CaCl_2_ structure was used for AIMD simulations. The hydrostatic structures at 130 GPa and 2000 to 4000 K were obtained by conducting simulations under *NPT* (*N*, number of particles; *P*, pressure; and *T*, temperature) ensemble with a time step of 1 fs for a total of 20 ps. Then the self-diffusion behavior was calculated using canonical ensemble (*NVT*) with a time step of 1 fs for a total of 20 ps. Langevin and Nosé thermostats were used for *NPT* and *NVT* simulations, respectively. The time-averaged mean square displacements (MSDs) of Al^3+^, H^+^, and O^2−^ were calculated using the atomic configurations from each simulation time step using Eq. 1

$$\langle {\left[\vec{r}\left(t\right)\right]}^{2}\rangle =\frac{1}{N}\sum_{i=1}^{N}\langle {\left[\vec{r_{i}}\left(t+t_{0}\right)-\vec{r_{i}}\left(t_{0}\right)\right]}^{2}\rangle$$ (1)

where $\vec{r_{i}}\left(t\right)$ is the displacement of the ith ion at time *t*, and *N* is the total number of protons in the system. In practice, *D* is obtained by a linear fit to the time dependence of the average MSDs.

$$D=\underset{0\to \infty }{\text{lim}}\left[\frac{1}{2\mathrm{dt}}\langle {\left[\vec{r}\left(t\right)\right]}^{2}\rangle \right]$$ (2)

In SI state, part of ions (H^+^ and Al^3+^ in this study) in the lattice present significant self-diffusion with MSDs increasing with simulation time, and other part of ions (O^2−^) vibrates at their lattice sites with insignificant MSDs increasement during the simulation.

### Deep potential training method and DFT calculation

The pretraining of the attention-based model (DPA-1) (*66*), implemented in the DeePMD-kit package (version 2.2.7) (*66*–*69*), was used for DP model training. An active learning procedure using the deep potential generator (DP-GEN) package (*70*) efficiently generated training datasets through an iterative exploration process. Six different structures, including δ-AlOOH, AlOOH (liquid), Al_2_O_3_ (Ru_2_O_3_-II structure), Ice X (bcc), Ice XVIII (fcc), and liquid water, were adopted in the DP-GEN iterations to calculate their energies, forces, and stresses for the training database. These iterations were conducted at temperatures ranging from 1000 to 5000 K and pressures from 60 to 160 GPa. In total, 10,006 configurations were selected for model training, and 388 configurations were randomly selected for model testing. During DP-GEN iterations, the cutoff radius was set to 6 Å, and the inverse distance 1/*r* decayed smoothly from 0.5 to 6 Å to eliminate discontinuities introduced by the cutoff. The sizes of the embedding network and fitting network were {25, 50, 100} and {240, 240, 240}, respectively. The production model used the same training parameters as the DP-GEN iterations, except that the smooth cutoff was adjusted from 0.5 to 10 Å to capture long-range interactions.

All DFT labeling for deep potential training was performed using ABACUS (version 3.6.0) with a linear combination of atomic orbitals basis set (*71*, *72*). The calculations used double-zeta plus polarization numerical atomic orbitals and norm-conserving pseudopotentials (SG15 ONCV) (*73*). The PBE functional (*65*) was applied, along with Grimme’s D3 dispersion correction (*74*). Atom counts for the DFT labeling were 64 for AlOOH, 80 for Al_2_O_3_, 162 for Ice X, 96 for Ice XVIII, and 192 for liquid water. The kinetic energy cutoff was set at 100 Ry, with an self-consistent field (SCF) convergence threshold for the density error of 1 × 10^−6^ Ry, and a K-spacing of 0.2.

In addition, four extra AIMD simulations were conducted on the AlOOH system at 1500, 2500, 3500, and 4000 K using the *NVT* ensemble. The K-spacing was set to 0.2, with a time step of 0.3 fs for thermalization. Each system was composed of 192 atoms. The initial configurations were obtained at 100 GPa at the respective temperatures of interest.

The accuracy of the DP model was initially evaluated by comparing its predictions to DFT calculations. Figures S8 and S9 show the comparison of force and energy for the test datasets. The overall prediction error in force values is 333 meV Å^−1^. The energy prediction errors are 8.08, 2.59, and 7.17 meV atom^−1^ for the AlOOH, Al_2_O_3_, and ice (ice X, ice XVIII, and liquid) systems, respectively. The comparison of radial distribution functions between DPMD and AIMD, illustrated in fig. S10, shows a close agreement. This demonstrates the convergence of the DP model, indicating that the trained DP model achieves an accuracy comparable to that of DFT.

### DPMD simulation and Gibbs free energy calculation

Using the DP model, we conducted DPMD simulations using the Large-scale Atomic/Molecular Massively Parallel Simulator package (*75*). Temperature and pressure were controlled using a Langevin thermostat (*76*) and a Nosé-Hoover barostat (*77*), respectively. A time step of 0.3 fs was used. The volume of the simulation cells was determined from 12 ps of DPMD simulations within *NPT* ensemble at the desired temperature and pressure, using the average box size from the latter half of the simulation. In δ-AlOOH, the calculated *P*-*V*-*T* relations present good consistence with the values calculated using AIMD simulation (fig. S4). We calculated Gibbs free energies of ice and AlOOH (solid, SI, and liquid phases) at pressures ranging from 60 to 160 GPa and temperatures between 1800 and 4600 K using the NeTI method (*41*, *78*–*80*) (see the Supplementary Materials for details).

## Supplementary Materials

### This PDF file includes:

Supplementary Text Figs. S1 to S21 Tables S1 to S3 References
