# Supplementary material for: Absence of dehydration due to superionic transition at Earth’s core-mantle boundary
Source: Sci Adv. 2026 Jan 28;12(5):eaeb3006. doi: 10.1126/sciadv.aeb3006 (PMC12851033; doi:10.1126/sciadv.aeb3006)
Supplement: Supplementary file 1 — Supplementary Text Figs. S1 to S21 Tables S1 to S3 References [file sciadv.aeb3006_sm.pdf]

Science Advances
AAAS

Supplementary Materials for

Absence of dehydration due to superionic transition at Earth's core-mantle boundary

Yu He *et al.*

Corresponding author: Yu He, heyu@mail.gyig.ac.cn

Sci. Adv. **12**, eaeb3006 (2026)
DOI: 10.1126/sciadv.aeb3006

This PDF file includes:

Supplementary Text
Figs. S1 to S21
Tables S1 to S3
References

Supplementary Text

Diffusion coefficient and ionic conductivity of δ-AlOOH

We conducted AIMD simulations on δ-AlOOH with different time steps of 0.5 and 1.0 fs and a larger supercell containing 512 atoms. We observed the diffusion of Al^{3+} in these simulations (fig. S11-S13). However, the calculated MSDs present significant differences. It suggests that the sampling efficiency of Al^{3+} diffusion in AIMD simulation is quite low, resulting in significant statistical errors (*84*). Therefore, we adopted deep potential molecular dynamics (DPMD) method, which has been proven to enable accurate calculation of diffusion coefficients by expanding the supercell and extending the simulation time (*85*).

We conducted DPMD simulations using the Large-scale Atomic/Molecular Massively Parallel Simulator (LAMMPS) package (*78*). Temperature and pressure were controlled using a Langevin thermostat (*79*) and a Nosé–Hoover barostat (*80*), respectively. A time step of 0.3 fs was employed. The volume of the simulation cells was determined from 12 ps of DPMD simulations within *NPT* ensemble at the desired temperature and pressure, using the average box size from the latter half of the simulation. The calculated *P-V-T* relations present good consistency with the values calculated using AIMD simulation (fig. S4). Then DPMD simulations with *NVT* ensemble were conducted at 70-130 GPa and 2000-4000 K for 15 ps using a large supercell containing 3456 atoms. The MSDs of H^{+}, Al^{3+}, and O^{2-} were calculated (fig. S5). As shown in fig. S14, the calculated MSDs are consistent with previous studies (*32*). Results from a larger supercell (8912 atoms) and longer simulation time (100 ps) also show consistency (Table S1). The MSDs present clear increasement with simulation temperature in larger simulation cell. Superionic transition leads to significant diffusion of H^{+} and Al^{3+} with increasing temperature, while the MSDs of O^{2-} do not show significant increasement during the simulation. Diffusion coefficients of H^{+}, Al^{3+} were calculated and shown in Fig. 3. The diffusion coefficients calculated at various temperatures were fitted with an Arrhenius equation:

$$D = \mathrm{A}\exp\left(-\frac{\Delta\mathrm{H}}{\mathrm{kT}}\right) \qquad (1)$$

where ΔH is the activation enthalpy, A is a pre-exponential factor, *k* is the Boltzmann constant, and *T* is the temperature. The calculated ΔH values for H^+ are 2.45, 2.60, 2.66, and 2.82 eV at 70, 90, 110, and 130 GPa, respectively. These values increases with pressure, suggesting that the presence of pressure hinders ionic diffusion, which is consistent with previous studies (*38*). It is the reason that superionic transition temperatures increase with applied pressure (Fig. 4). The electrical conductivities contributed by ionic diffusion were calculated using the Nernst-Einstein equation:

$$\sigma = \frac{\mathrm{fD}cq^2}{kT} \qquad (2),$$

in which σ is the electrical conductivity, *f* is a numerical factor approximately equal to unity, *D* is the diffusion coefficient, *c* is the concentration, *q* is the electrical charge of H^+ and Al^{3+}, *k* is the Boltzmann constant, and *T* is the temperature.

Ice phase diagram calculation

Superionic ice was observed in high pressure and high temperature experiments (*24-27*), and liquid like protons contribute external entropy leading to phase transition from bcc to fcc structure. Superionic phases are important to understand the structure and dynamics of icy giants' interiors (*86-89*). The stability of superionic ice is also important for the dehydration process in Earth's interior. Thus, we calculated Gibbs free energies of ice at pressures ranging from 30 to 160 GPa and temperatures between 1800 and 4400 K using the nonequilibrium thermodynamic integration (NeTI) method (*81-83*). The NeTI method has been widely utilized to investigate the melting and phase transition behaviors of various materials (*90-92*).

Generally, the free energy of a thermal equilibrium system with *N* particles, a volume *V* at temperature *T* can be expressed as:

$$F\,(N,V,T;\lambda) = -k_B \mathrm{T} \ln\left[\frac{1}{\Lambda^{3N} N!}\right] \int_V \exp\left(-\frac{H(\boldsymbol{r},\lambda)}{k_B \mathrm{T}}\right) d\boldsymbol{r} \qquad (3),$$

where, Λ is the thermal de Broglie wavelength, *V* denotes the volume, $H(\boldsymbol{r},\lambda)$ is the Hamiltonian of the system under a particular value of λ. In comparison with energy different between two systems characterized by different λ values (namely λ_i and λ_f), the free energy difference can be expressed as

$$\Delta F = F(\lambda_i) - F(\lambda_f) = \int_{\lambda_i}^{\lambda_f} d\lambda \frac{\partial F}{\partial \lambda} = \int_{\lambda_i}^{\lambda_f} d\lambda \left\langle \frac{\partial H}{\partial \lambda} \right\rangle_{\lambda} \quad (4),$$

where the angular bracket is the canonical ensemble average for λ. The integral can also be expressed as $W_{i\to f}^{rev}$, which suggests the reversible work along a quasistatic process between two thermodynamic conditions. In a standard thermodynamic integration (TI) approach, free energy difference can be determined by carrying out a series of independent equilibrium simulations of $H(\boldsymbol{r}, \lambda)$ on a grid of λ between λ_i and λ_f.

On the other hand, nonequilibrium method offers notable efficiency advantages (*41, 81-83*). In NeTI, a time-dependent parameter $\lambda = \lambda(\mathrm{t})$, which varies between λ_i and λ_f, is adopted in the simulation. The irreversible work throughout the simulation can be expressed as

$$W_{i\to f}^{irr} = \int_0^{\mathrm{t}_s} d\mathrm{t} \frac{d\lambda}{dt} \left\langle \frac{\partial H}{\partial \lambda} \right\rangle_{\Gamma(\mathrm{t})} \quad (5),$$

where $\Gamma(\mathrm{t})$ denotes the phase-space trajectory at time *t* for a total simulation duration of t_s. In the context of the quasistatic nonequilibrium process with long simulation time (t_s), the system bias can be mitigated by integrating along two opposing directions, allowing for the calculation of free energy difference:

$$\Delta F = F(\lambda_i) - F(\lambda_f) = \frac{1}{2}\left[W_{i\to f}^{rev} - W_{f\to i}^{rev}\right] = \frac{1}{2}\left[\overline{W_{\iota\to f}^{\iota rr}} - \overline{W_{f\to \iota}^{\iota rr}}\right] \quad (6).$$

For the superionic phase, the Gibbs free energy was assessed using a two-step NeTI method (*41*). In this approach, the reference Gibbs free energy of a bulk system is derived from the individual contributions of the Einstein solid and Uhlenbeck-Ford fluid (UF):

$$F_{ref} = F_{Einstein} + F_{UF} \quad (7),$$

particular parametric Hamiltonian is in the form of

$$H(\lambda) = \lambda H_{SI} + (1 - \lambda) H_{ref} \quad (8),$$

where H_{SI} is the Hamiltonian for the superionic phase. Then the free energy of superionic water can be expressed as

$$F_{SI} = F_{ref} + \int_0^1 d\lambda \left\langle \frac{\partial H}{\partial \lambda} \right\rangle_{\lambda} = F_{ref} + \int_0^1 d\lambda \left\langle U_{SI} - U_{Einstein,O} - U_{UF,H} \right\rangle_{\lambda} \quad (9),$$

where U_{SI}, $U_{Einstein,O}$, and $U_{UF,H}$ represent interatomic potential of superionic ice, Einstein solid potential for solid-like oxygen, and Uhlenbeck-Ford fluid potential for liquid-like hydrogen. To prevent particle overlaps at λ = 0, which could lead to singular behavior in the driving force, an auxiliary system was introduced. This system incorporates an artificial repulsive UF interaction

term between the Einstein oscillators and the UF fluid particles. The Gibbs free energy for the superionic phase can be determined using:

$$G_{SI}(N,V,T) = F_{SI}^{ref}(N,V,T) + W_{ref\to aux}^{rev} + W_{aux\to SI}^{rev} + P(N,T)V(N) \quad (10).$$

Using the two-step NeTI method, Fidalgo Cândido et al. (*41*) successfully determined that the melting temperature of fcc water to liquid at 340 GPa is 6340 K. Their findings are consistent with earlier estimates (*24*).

The NeTI free energy simulations for water were conducted in an *NVT* ensemble. The volume of the simulation cells was determined from 12 ps of isothermal-isobaric MD simulations (*NPT*) at the desired temperature and pressure, using the average box size from the latter half of the simulation. The spring constants for the unmoving Einstein particles were derived from the mean-squared displacements (MSDs) of the corresponding particles during the *NPT* equilibrium. During the simulation process, the center of mass of the non-diffusing particle subsystem was held fixed (*81*). The UF interaction parameters for O-H and H-H were adopted from Fidalgo Cândido et al. (*41*).

We calculated the Gibbs free energy of water at pressures ranging from 40 to 140 GPa. The DPMD calculation systems consisted of 4374 atoms for ice X, 4116 atoms for ice XVIII, and 4500 atoms for liquid phase. At constant pressure, the Gibbs free energy is a function of temperature, allowing us to determine the phase transition temperature by finding the intersection of two curves. The phase transition temperatures between ice X and ice XVIII at 50, 60, 80, 100, 120, and 140 GPa are 1780, 2050, 2130, 2205, 2270, and 2305 K, respectively (fig. S15). The phase transition temperatures between superionic ice XVIII and liquid phase at 100, 120, and 140 GPa are 3150, 3604 and 4035 K, respectively (fig. S15). The phase transition points were collected in Table S2. The melting temperature at 100 GPa is close to the result of 3250 K reported by Cheng et al. (2021) (*28*).

Using the phase transition point at 100 GPa, we calculated the phase transition line using the dynamic Clausius–Clapeyron integration (dCCI) method (*83*) (fig. S1). The phase boundaries were independently calculated with the dCCI method. As shown in fig. S1, the boundary lines align well with the transition points, providing a double validation of our results. The calculated results are compared with previous experimental (*24-27*) and computational studies (*28*) in main manuscript Fig. 1. Generally, our results are consistent with previous results.

δ-AlOOH phase diagram calculation

Under deep lower mantle pressure, δ-AlOOH undergoes doubly superionic transition with increasing temperature as suggested by both AIMD and DPMD simulations (Fig. 2 and Fig. 3). The conditions of phase transition from solid phase to SI-I, and to SI-II were estimated based on the calculated diffusion coefficients (Fig. 3A and Fig. 4). The calculated transition temperatures for solid to SI-I transition are ~2000-2300 K at 70-130 GPa, and transition temperatures for SI-I to SI-II transition are ~3550-3950 K at 70-130 GPa. However, we cannot calculate melting temperature for SI-II based on the diffusion of oxygen due to superheating effect. To eliminate the influence of overheating state, we calculated the Gibbs free energies for solid, SI-I, SI-II, and liquid phases at 2000-4600 K and 60-140 GPa using NeTI method as described in the previous section. The simulation model contains 3456 atoms. The free energies of SI-I and SI-II can be expressed as

$$F_{SI-I} = F_{ref} + \int_0^1 d\lambda \langle U_{SI-I} - U_{Einstein,O} - U_{Einstein,Al} - U_{UF,H} \rangle_\lambda \qquad (11),$$

$$F_{SI-II} = F_{ref} + \int_0^1 d\lambda \, \langle U_{SI-II} - U_{Einstein,O} - U_{UF,Al} - U_{UF,H} \rangle_\lambda \qquad (12),$$

$U_{Einstein,Al}$ and $U_{UF,Al}$ indicate that Al is treated as Einstein solid potential in SI-I, and Uhlenbeck-Ford fluid potential in SI-II. The UF interaction parameters for O-H and H-H were adopted from Fidalgo Cândido et al. (*41*). The UF interaction parameter for Al-H was set to be the same as that for O-H.

The Gibbs free energies of solid phase, SI-I, SI-II, and liquid phase are presented in fig. S16 & fig. S17. As shown in fig. S17, at low temperatures, the free energy of solid phase is the lowest. At temperature above ~2200-2300 K, SI-I is the lowest. As the temperature increases, the stable phase transforms to SI-II suggesting that doubly superionic transition is energetically favorable in δ-AlOOH system. Liquid phase becomes energetically favorable at temperatures above 3800 K indicating the melting of the δ-AlOOH. It is worth noting that we compared the melting temperatures for SI-I and SI-II and found that the melting temperature for SI-II is higher than that of SI-I at pressures below ~138 GPa. It means that diffusion of Al^{3+} in the lattice indeed contributes external entropy to the system and stabilize the superionic phase. This result is the same as superionic ice that superionic transition is able to increase the melting temperature (*24-28*). At pressure above ~138 GPa, SI-I phase transform directly to liquid phase. The instability of SI-II may be attributed to the instability of Al-site vacancies with increasing pressure. The calculated phase diagram and phase transition conditions for δ-AlOOH is shown in Fig. 4 and Table S3.

Melting temperatures calculated by two phase coexisting simulations

To verify the reliability of two-step TI method, we calculated the melting temperatures of fcc SI H_2O and δ-AlOOH by conducting solid-liquid two phase coexisting DPMD simulations at 40-140 GPa and 1500-4100 K. The DPMD simulations were conducted on the two-phase model containing 12288 atoms for H_2O (fig. S18) and 6912 atoms for AlOOH (fig. S19) in the *NPT* ensemble with a time step of 0.3 fs for up to 100,000 steps. The energy variation of the solid-liquid two-phase system can directly and intuitively reflect the crystallization and melting processes. A decrease in energy indicates a crystallization process, while an increase in the energy indicates a melting process (fig. S20 and fig. S21). The states of systems were cross checked by the trajectories of the atoms. The calculated melting temperatures exhibit good agreement with the results obtained using two-step TI method (fig. S1 and fig. S6).

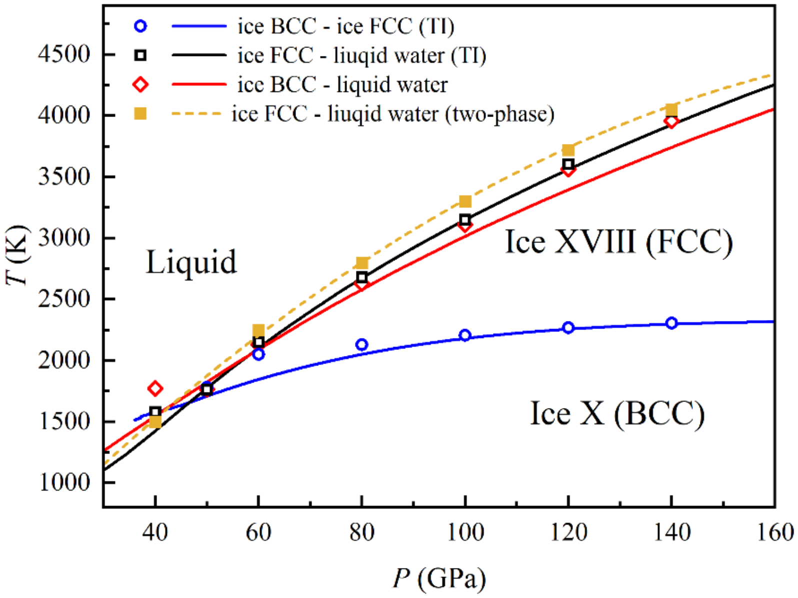

Fig. S1. The calculated phase diagram of H_2O ice at 30-160 GPa and 1000-4500 K using two-step TI and two-phase methods.

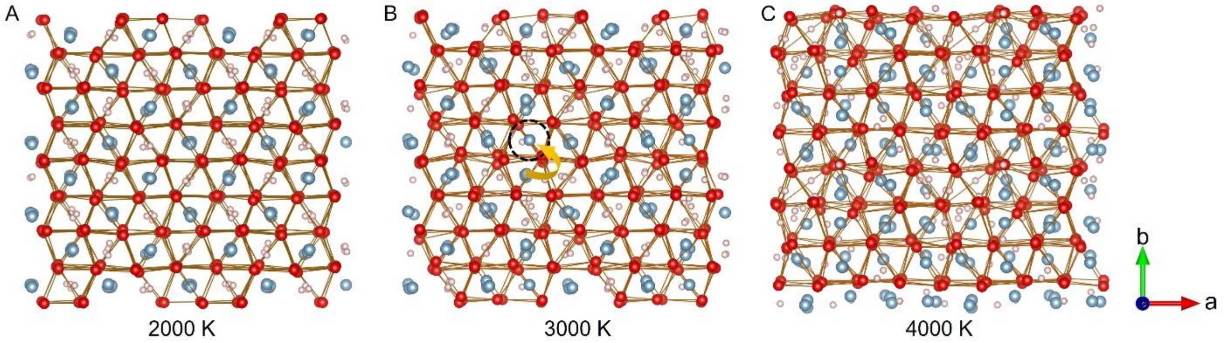

Fig. S2. The lattice structure of δ-AlOOH after 20 ps AIMD simulations at 130 GPa and temperatures of (A) 2000, (B) 3000, and (C) 4000 K. H, Al, and O are shown with light pink, light blue, and red spheres. The O sub-lattices (dark red lines) are stable after simulations. The yellow arrow (B) notes a Al^{3+} migration along b axis, observed in the simulation at 3000 K from the Al-site to H-site (dashed black circle) in δ-AlOOH.

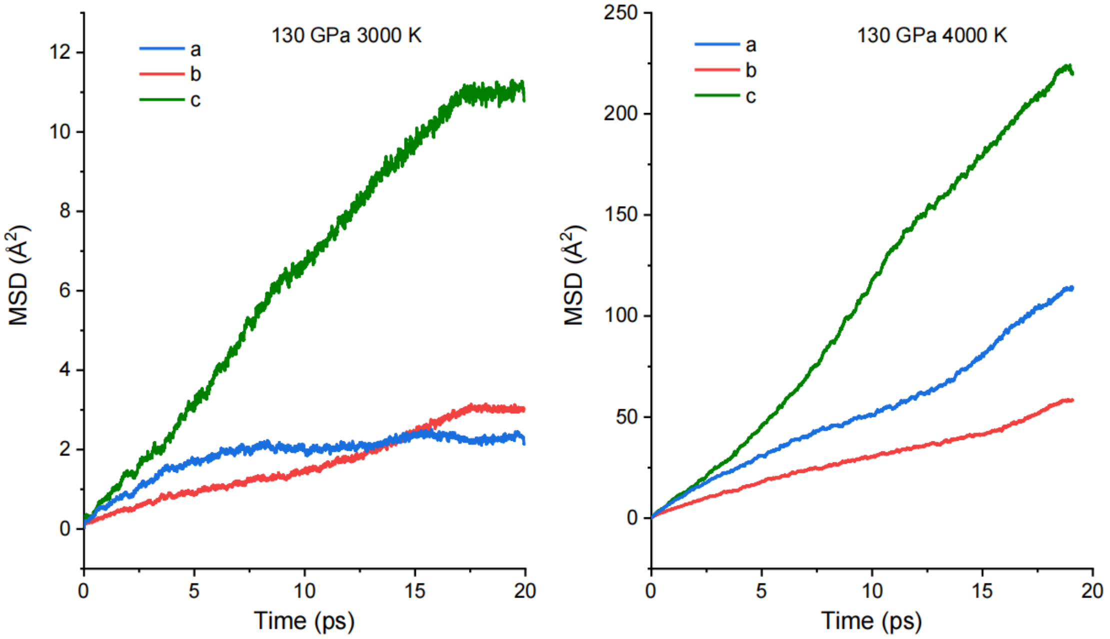

Fig. S3. MSDs of H^+ along a, b, and c lattice directions in δ-AlOOH at 130 GPa, and 3000 and 4000 K.

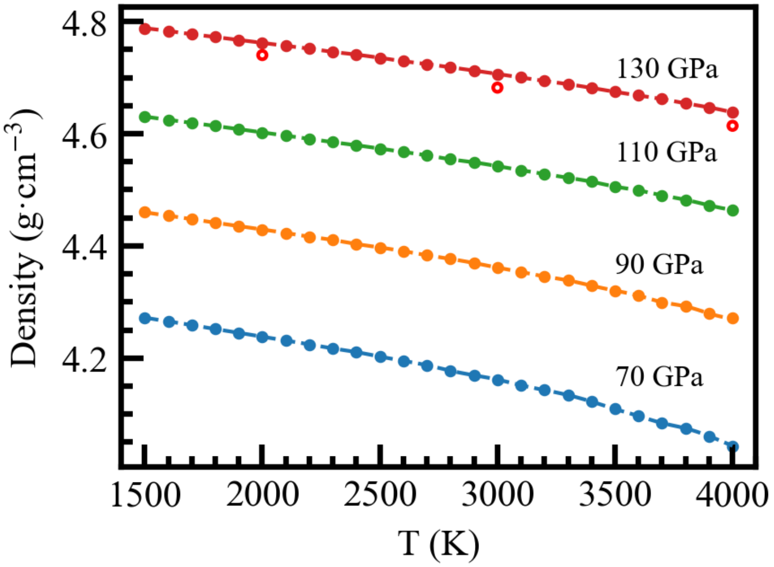

Fig. S4. Calculated density (*ρ*)-pressure (*P*)-temperature (*T*) rations of δ-AlOOH using DPMD method at 70-130 GPa and 1500-4000 K. The data at 130 GPa is compared with the results computed using AIMD simulations (empty red cycles).

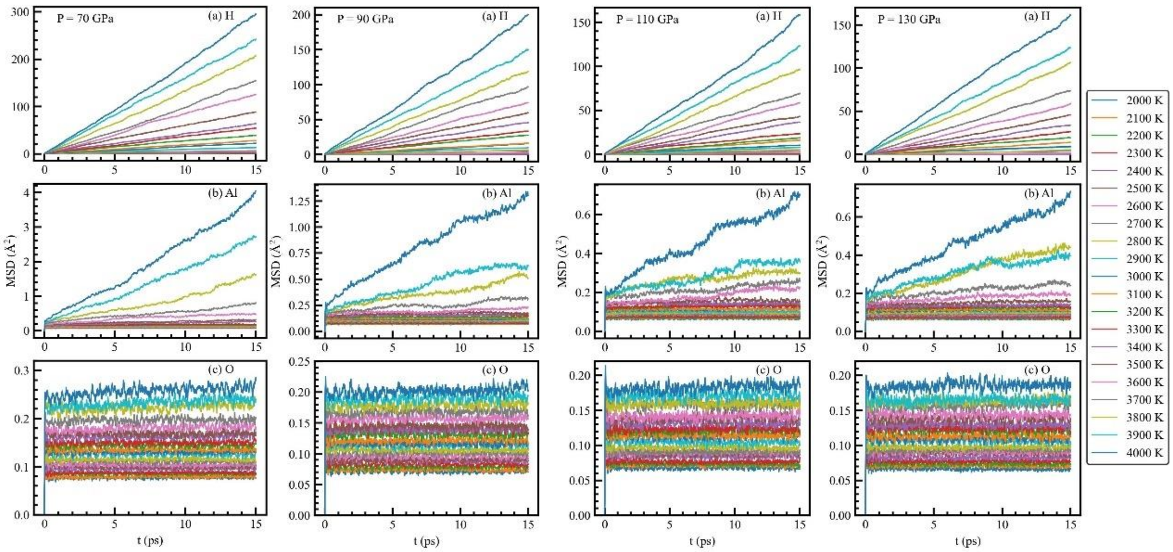

Fig. S5. The MSDs of H, Al, and O ions in δ-AlOOH at 70-130 GPa and 2000-4000 K.

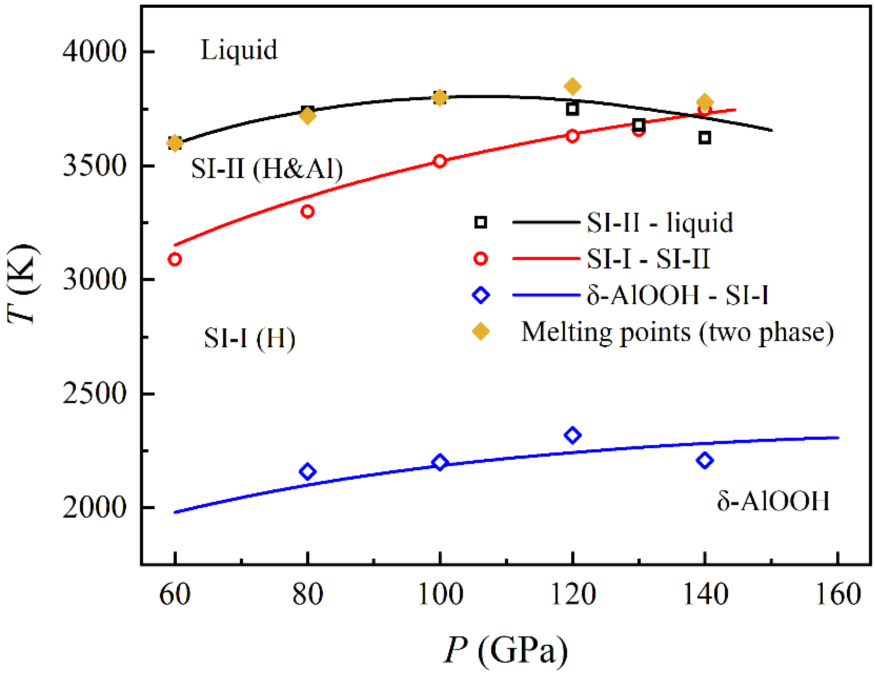

Fig. S6. The phase diagram of δ-AlOOH at 60-160 GPa and 2000-4000 K using two-step TI and two-phase methods.

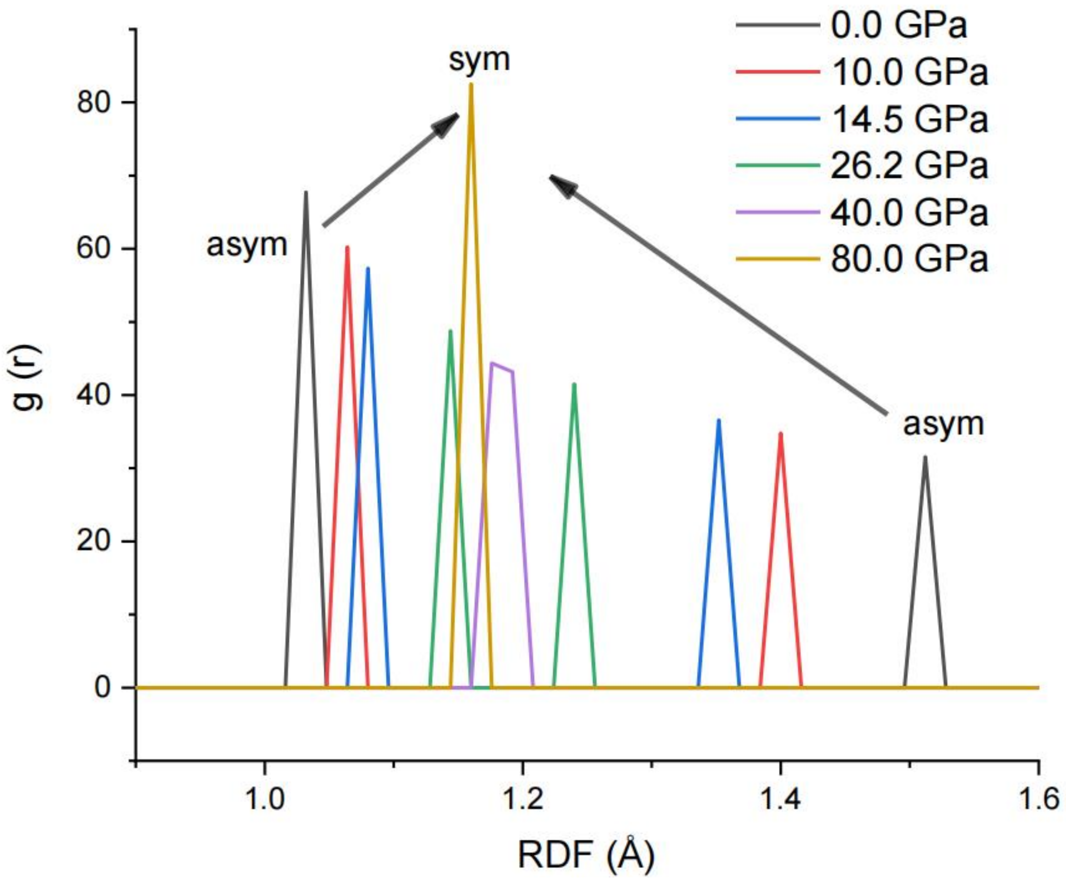

Fig. S7. Radial distribution functions (RDFs) of O-H in δ-AlOOH with increasing pressure. The asymmetric to symmetric O-H bonding transition takes place at pressure of ~40 GPa.

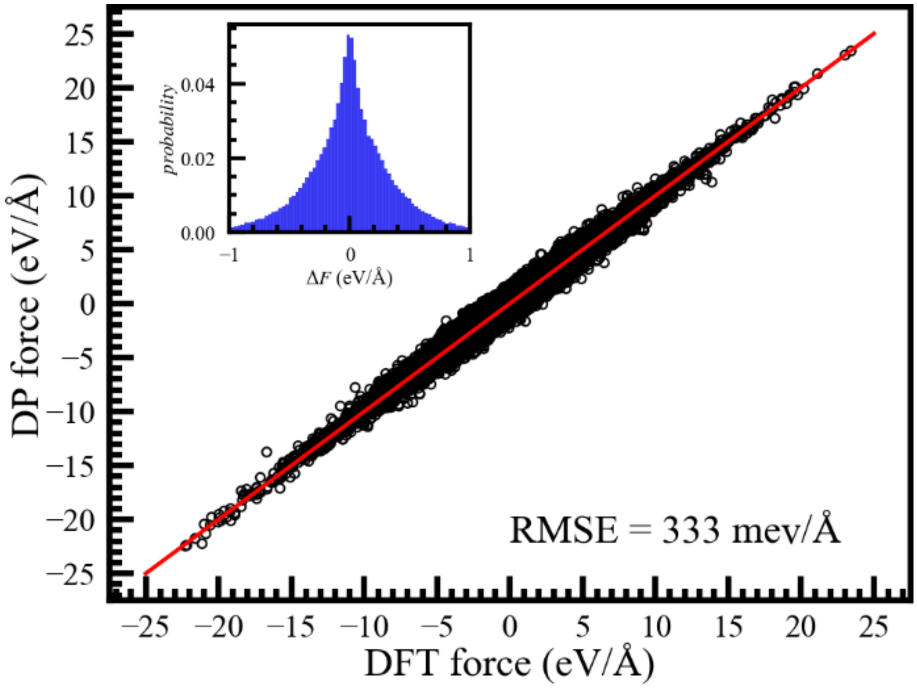

Fig. S8. Comparison of force predictions between the DP model and DFT calculations of configurations from the test data sets.

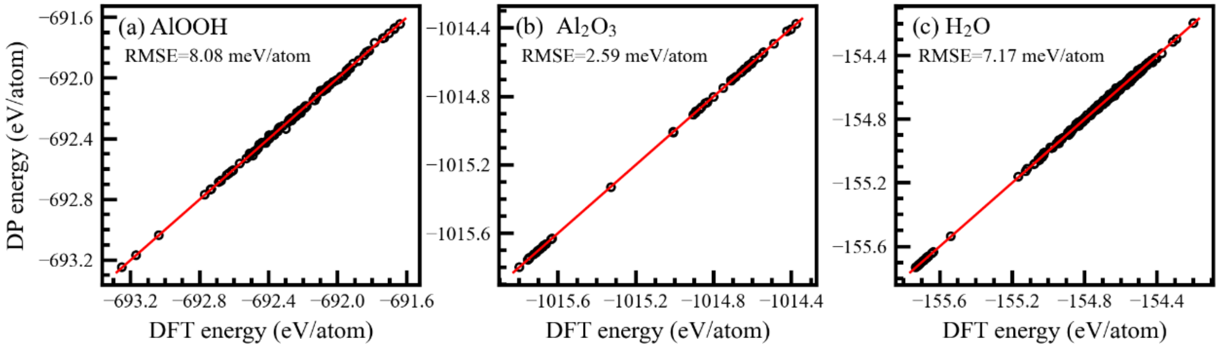

Fig. S9. Comparison of energy predictions between the DP model and DFT calculations of configurations from the test data sets.

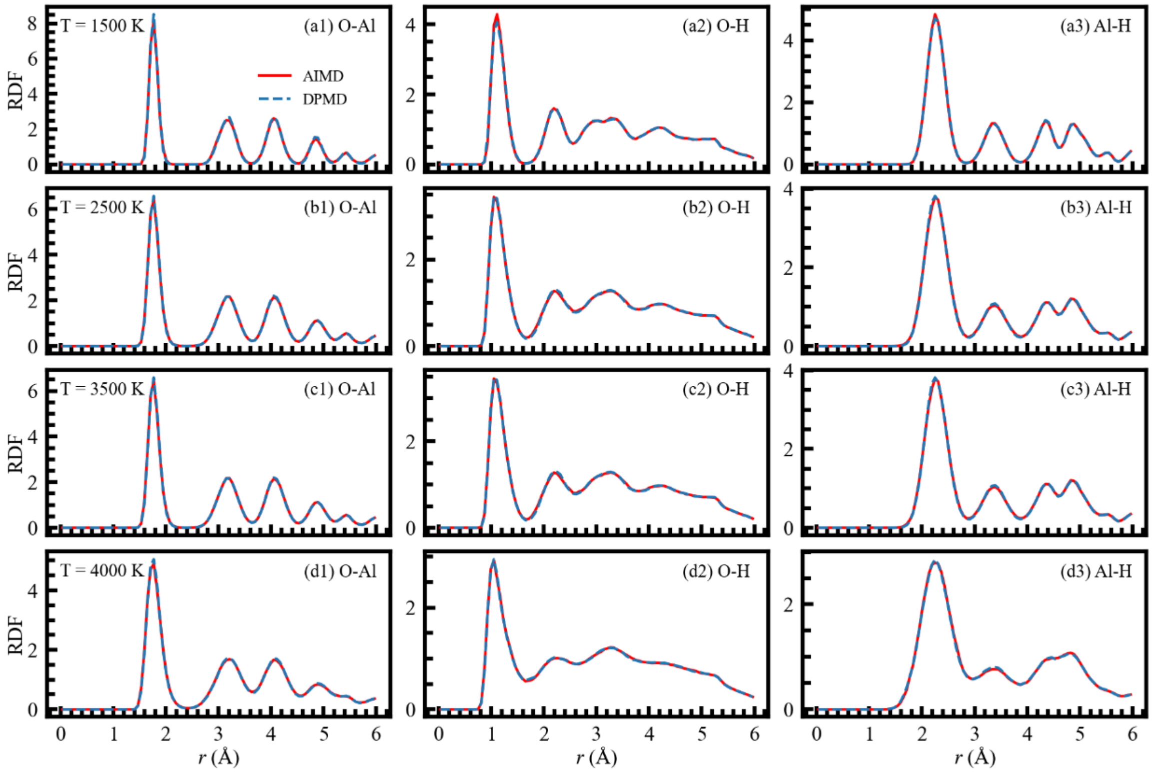

Fig. S10. Comparison of the RDFs obtained from AIMD and DPMD methods for AlOOH at 1500 K, 2500 K, 3500 K and 4000 K under 100 GPa.

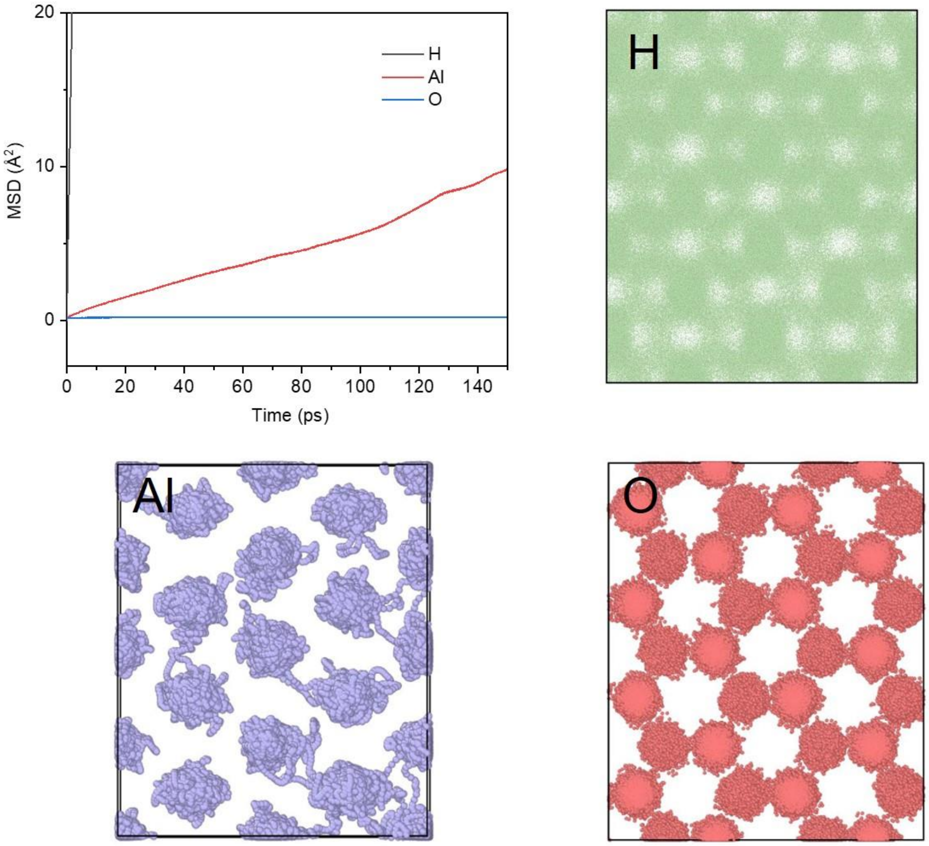

Fig. S11. AIMD calculated MSDs and trajectories (bc-plane) of H^+, Al^{3+} and O^{2-} in δ-AlOOH at 130 GPa and 4000 K with a supercell containing 128 atoms and a time step of 0.5 fs.

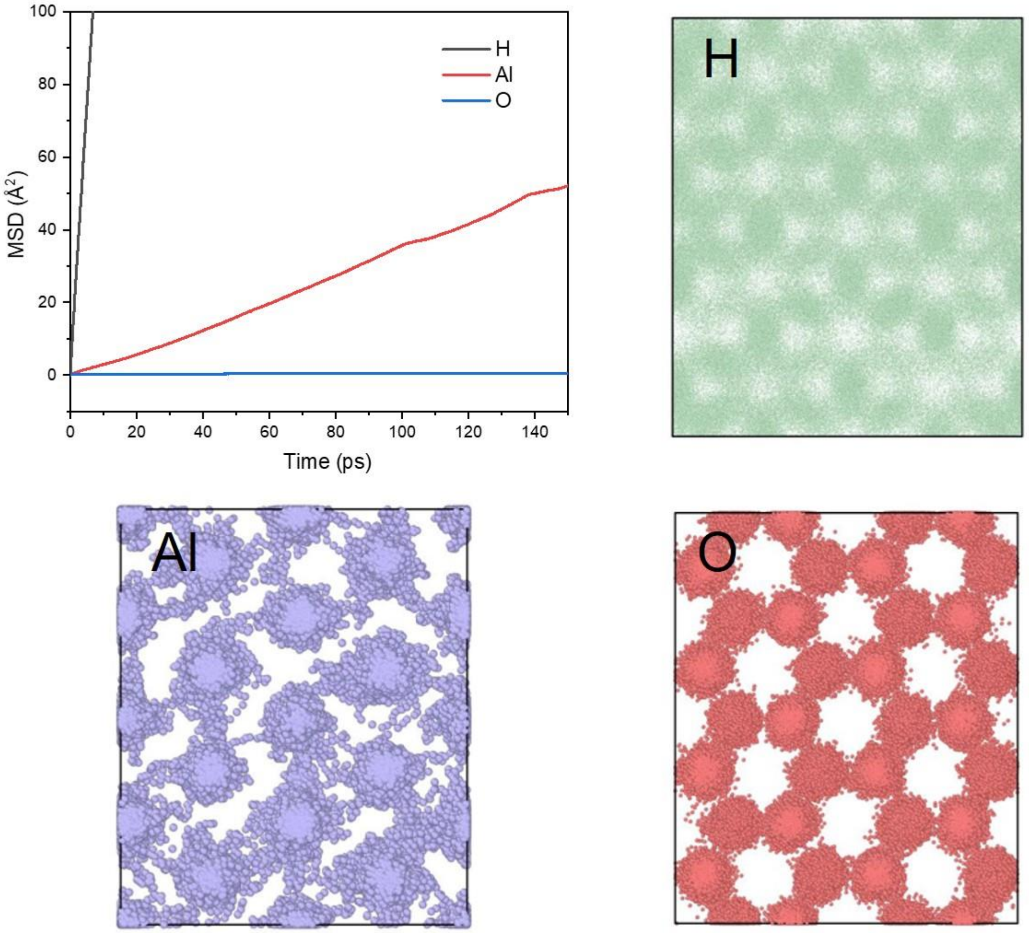

Fig. S12. AIMD calculated MSDs and trajectories (bc-plane) of H^+, Al^{3+} and O^{2-} in δ-AlOOH at 130 GPa and 4000 K with a supercell containing 128 atoms and a time step of 1.0 fs.

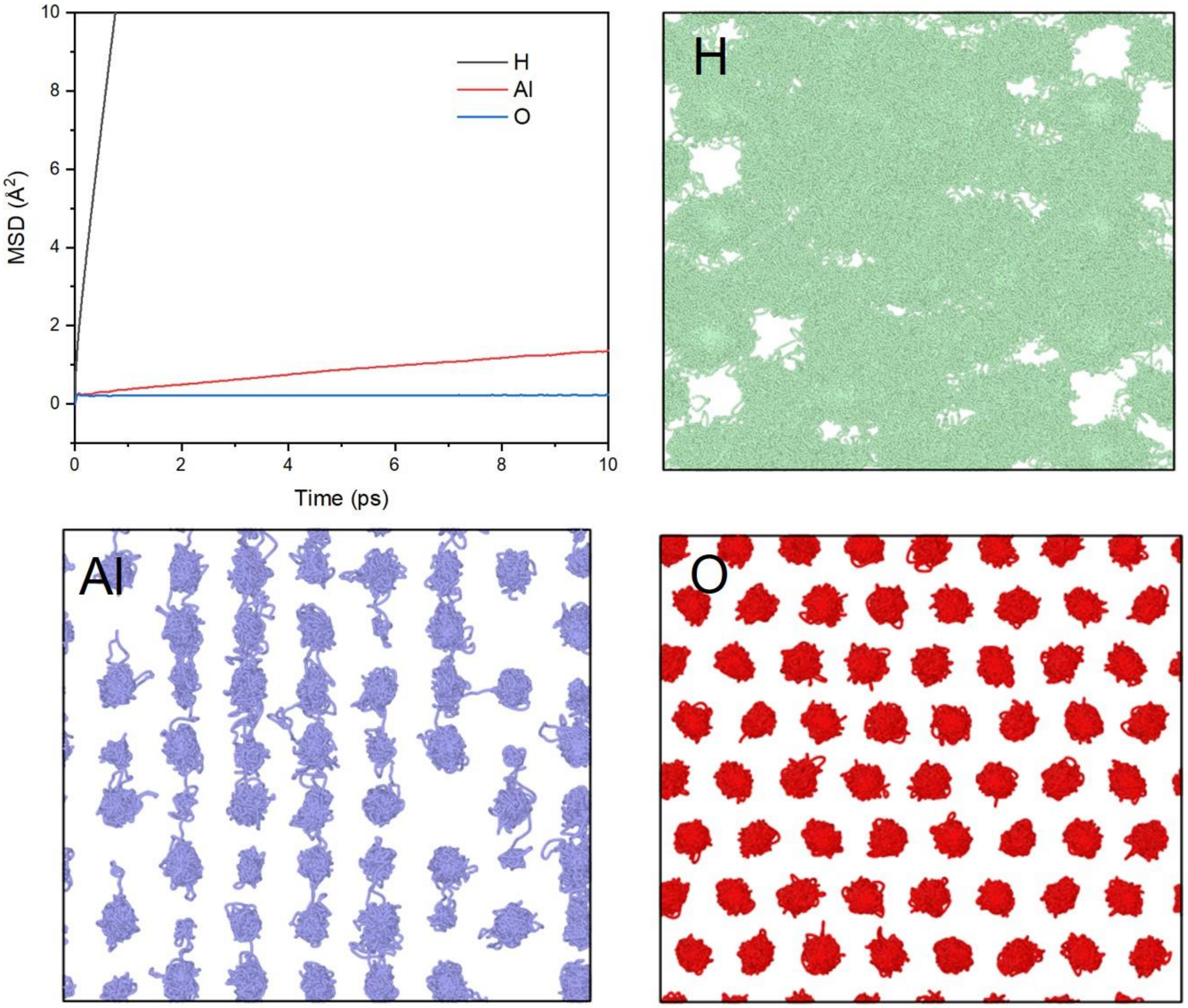

Fig. S13. AIMD calculated MSDs and trajectories (bc-plane) of H^+, Al^{3+} and O^{2-} in δ-AlOOH at 60 GPa and 3500 K with a supercell containing 512 atoms and a time step of 1.0 fs.

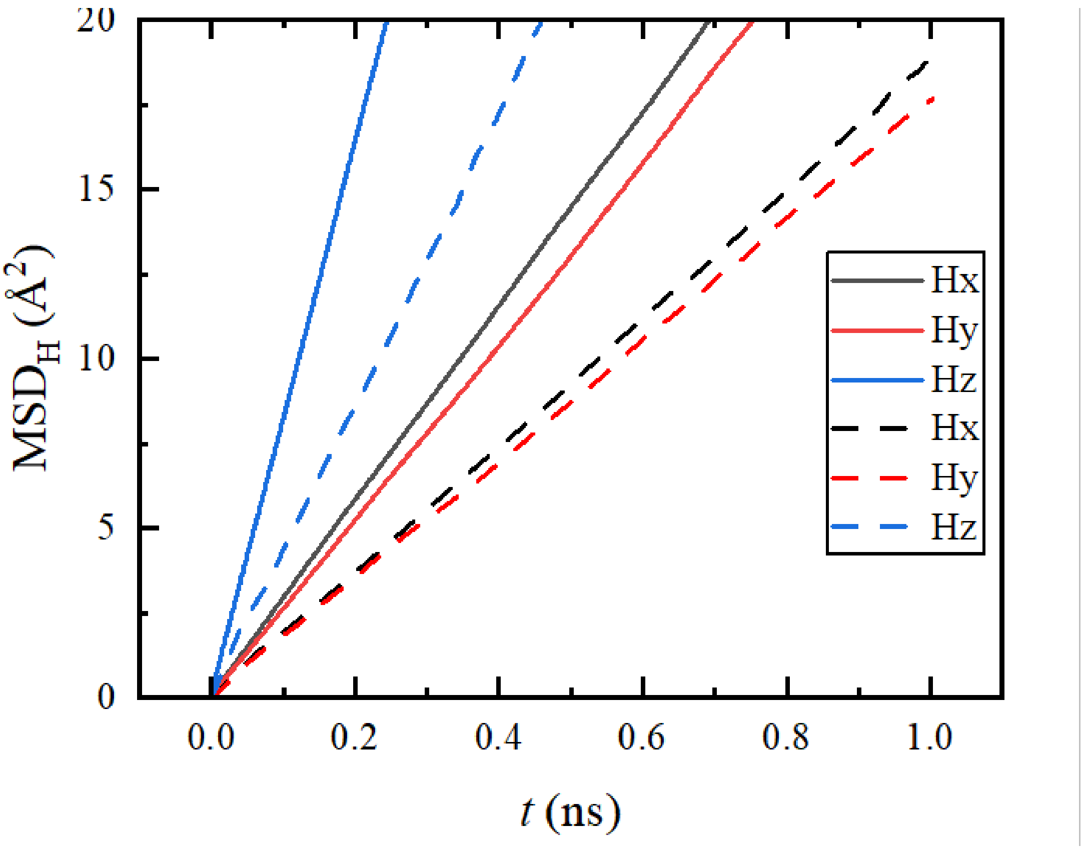

Fig. S14. Calculated MSDs along different lattice directions in δ-AlOOH at 67 GPa and 2400 K. The results (dashed lines) are compared with the results of previous study (*6*) under the same conditions.

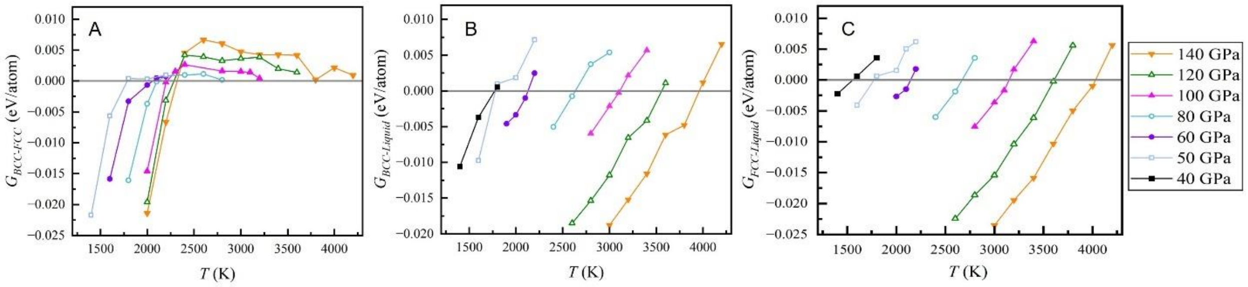

Fig. S15 The Gibbs free energies differences between different H_2O phases. (**A**) ice X (bcc) and ice XVIII (fcc, superionic), (**B**) ice X (bcc) and liquid water and (**C**) ice XVIII (fcc, superionic) and liquid water as a function of temperature at different pressures.

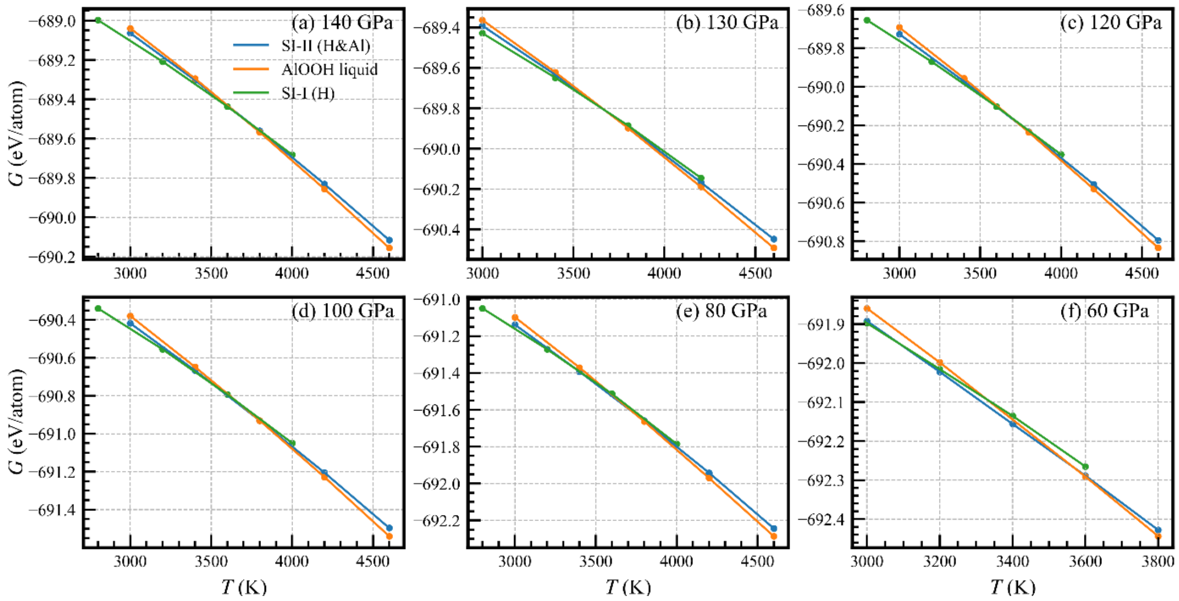

Fig. S16. The change of Gibbs free-energy of solid, SI-I, SI-II and liquid AlOOH as a function of temperature at 80-140 GPa.

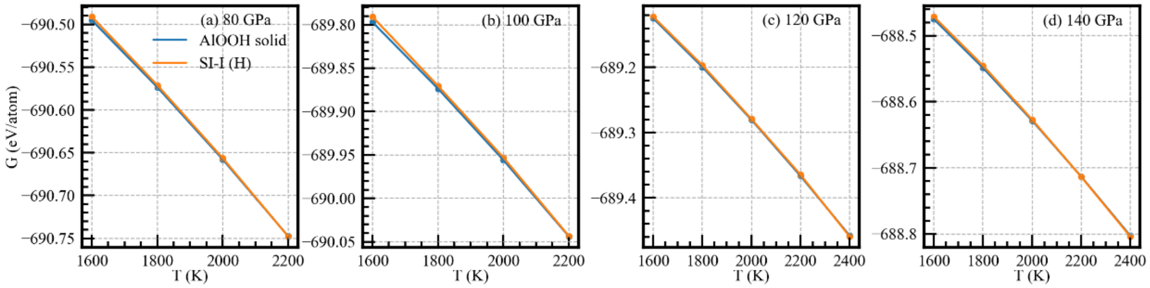

Fig. S17. The change of Gibbs free energies of solid and SI-I δ-AlOOH as a function of temperature at ~80-140 GPa.

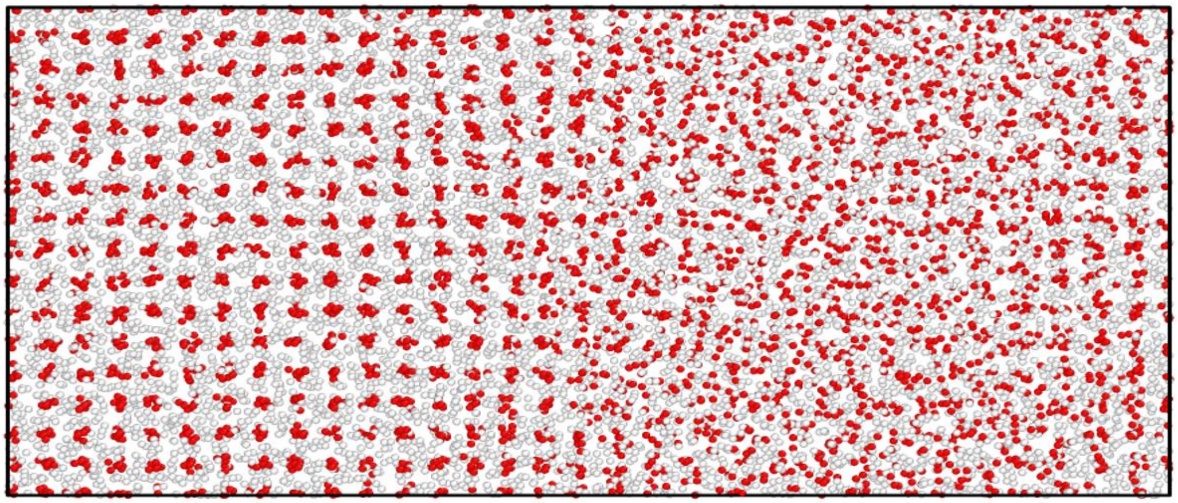

Fig. S18. Two phase coexisting structure of fcc SI H_2O and liquid H_2O for DPMD simulations. H and O atoms are shown with white and red spheres.

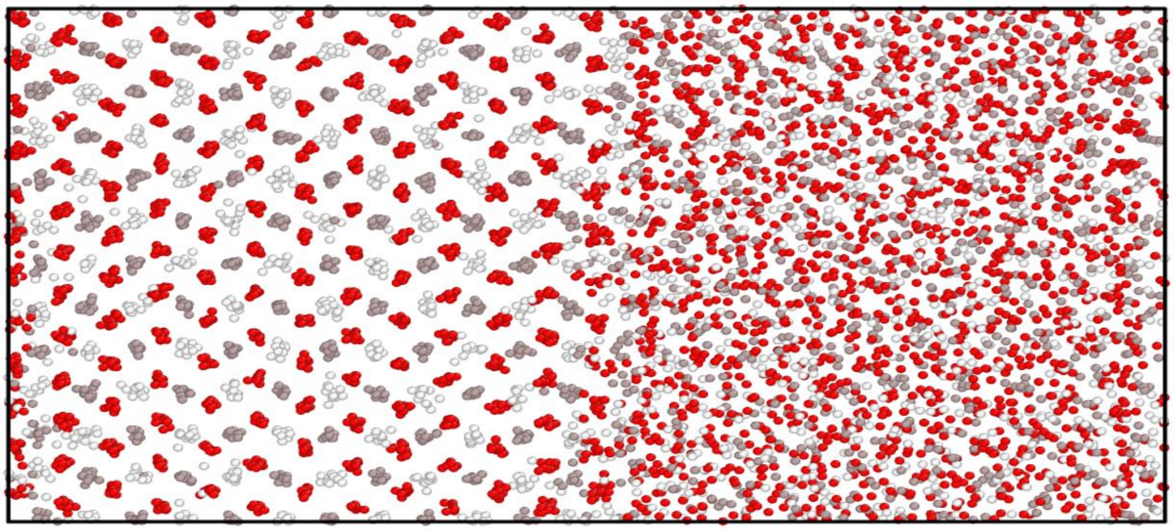

Fig. S19. Two phase coexisting structure of δ-AlOOH and liquid AlOOH for DPMD simulations. H, O, and Al atoms are shown with white, red, and grey spheres.

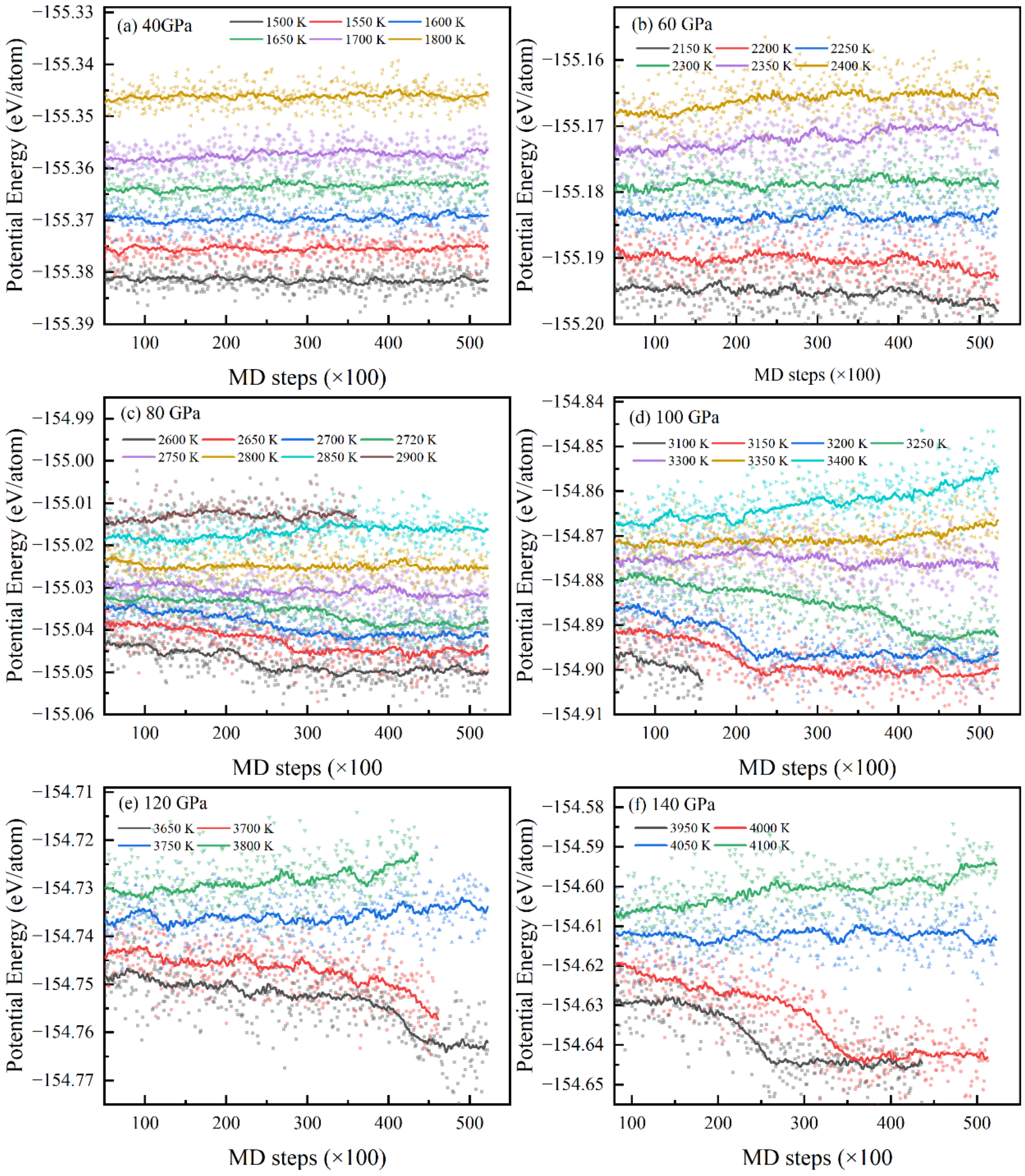

Fig. S20. Potential energy per atom versus time for the fcc H_2O solid-liquid two-phase system under varying temperatures and pressures from *NPT* simulations.

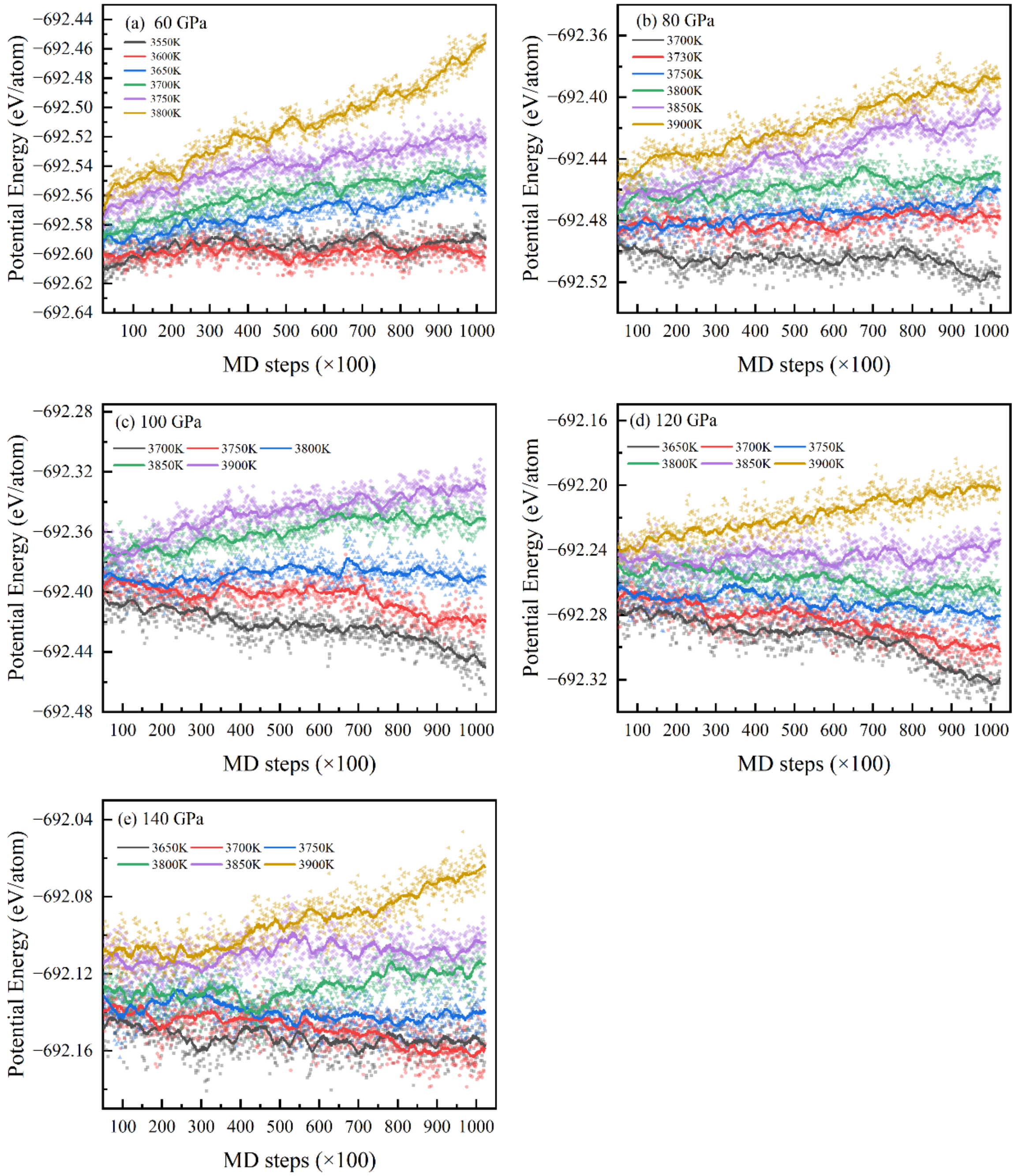

Fig. S21. Potential energy per atom versus time for the AlOOH solid-liquid two-phase system under varying temperatures and pressures from *NPT* simulations.

Table S1. Comparison of calculated diffusion coefficients of H^+ and Al^{3+} using different supercells and simulation times.

		8912atoms, 100 ps, m^2/s		3456 atoms, 15 ps, m^2/s	
T/K	P/GPa	D_{Al}	D_H	D_{Al}	D_H
4000	90	3.59E-10	2.95E-8	2.84E-10	2.67E-8

Table S2. The phase transition temperatures and pressures of water.

P (GPa)	T (K) ice X->ice XVIII	T (K) ice XVIII->liquid	T (K) ice X->liquid
40	–	1580	1772
50	1780	1760	1768
60	2050	2150	2130
80	2130	2680	2630
100	2205	3150	3110
120	2270	3604	3565
140	2305	4035	3960

Table S3. The phase transition temperatures and pressures of AlOOH.

P (GPa)	T (K) δ-AlOOH->SI-II	T (K) SI-I->SI-II	T (K) SI-II->liquid	T (K) SI-I->liquid
60	–	3090	3600	3350
80	2160	3300	3735	3580
100	2200	3520	3800	3670
120	2320	3630	3750	3685
130	–	3658	3680	3668
140	2210	3747	3624	3689

REFERENCES

1. Q. Williams, R. J. Hemley, Hydrogen in the deep Earth. *Annu. Rev. Earth Planet. Sci.* **29**, 365–418 (2001).

2. E. Ohtani, The role of water in Earth's mantle. *Natl. Sci. Rev.* **7**, 224–232 (2020).

3. E. Ohtani, Hydration and dehydration in Earth's interior. *Annu. Rev. Earth Planet. Sci.* **49**, 253–278 (2021).

4. A. V. Milkov, Molecular hydrogen in surface and subsurface natural gases: Abundance, origins and ideas for deliberate exploration. *Earth-Sci. Rev.* **230**, 104063 (2022).

5. E. Hand, Hidden hydrogen *Science* **379**, 630–636 (2023).

6. M. Nishi, T. Irifune, J. Tsuchiya, Y. Tange, Y. Nishihara, K. Fujino, Y. Higo, Stability of hydrous silicate at high pressures and water transport to the deep lower mantle. *Nat. Geosci.* **7**, 224–227 (2014).

7. E. Ohtani, Y. Amaike, S. Kamada, T. Sakamaki, N. Hirao, Stability of hydrous phase H $MgSiO_4H_2$ under lower mantle conditions. *Geophys. Res. Lett.* **41**, 8283–8287 (2014).

8. A. Sano, E. Ohtani, T. Kondo, N. Hirao, T. Sakai, N. Sata, T. Kikegawa, Aluminous hydrous mineral δ-AlOOH as a carrier of hydrogen into the core–mantle boundary. *Geophys. Res. Lett.* **35**, 303 (2008).

9. I. Ohira, E. Ohtani, T. Sakai, M. Miyahara, N. Hirao, Y. Ohishi, M. Nishijima, Stability of a hydrous δ-phase, AlOOH–$MgSiO_2(OH)_2$, and a mechanism for water transport into the base of lower mantle. *Earth Planet. Sci. Lett.* **401**, 12–17 (2014).

10. Y. Duan, N. Sun, S. Wang, X. Li, X. Guo, H. Ni, V. B. Prakapenka, Z. Mao, Phase stability and thermal equation of state of δ-AlOOH: Implication for water transportation to the deep lower mantle. *Earth Planet. Sci. Lett.* **494**, 92–98 (2018).

11. H. Piet, K. D. Leinenweber, J. Tappan, E. Greenberg, V. B. Prakapenka, P. R. Buseck, S. H. Shim, Dehydration of δ-AlOOH in Earth's deep lower mantle. *Minerals* **10**, 384 (2020).

12. Q. Hu, D. Y. Kim, W. Yang, L. Yang, Y. Meng, L. Zhang, H. K. Mao, FeO_2 and FeOOH under deep lower-mantle conditions and Earth's oxygen–hydrogen cycles. *Nature* **534**, 241–244 (2016).

13. M. Nishi, Y. Kuwayama, J. Tsuchiya, T. Tsuchiya, The pyrite-type high-pressure form of FeOOH. *Nature* **547**, 205–208 (2017).

14. Q. Hu, D. Y. Kim, J. Liu, Y. Meng, L. Yang, D. Zhang, W. L. Mao, H. K. Mao, Dehydrogenation of goethite in Earth's deep lower mantle. *Proc. Natl. Acad. Sci. U.S.A.* **114**, 1498–501 (2017).

15. J. Liu, Q. Hu, D. Young Kim, Z. Wu, W. Wang, Y. Xiao, P. Chow, Y. Meng, V. B. Prakapenka, H.-K. Mao, W. L. Mao, Hydrogen-bearing iron peroxide and the origin of ultralow-velocity zones. *Nature* **551**, 494–497 (2017).

16. Y. Lin, Q. Hu, Y. Meng, M. Walter, H. K. Mao, Evidence for the stability of ultrahydrous stishovite in Earth's lower mantle. *Proc. Natl Acad. Sci. U.S.A.* **117**, 184–189 (2020).

17. C. Nisr, H. Chen, K. Leinenweber, A. Chizmeshya, V. B. Prakapenka, C. Prescher, S. N. Tkachev, Y. Meng, Z. Liu, S. H. Shim, Large H_2O solubility in dense silica and its implications for the interiors of water-rich planets. *Proc. Natl Acad. Sci. U.S.A.* **117**, 9747–9754 (2020).

18. T. Ishii, G. Criniti, E. Ohtani, N. Purevjav, H. Fei, T. Katsura, H.-K. Mao, Superhydrous aluminous silica phases as major water hosts in high-temperature lower mantle. *Proc. Natl Acad. Sci. U.S.A.* **119**, e2211243119 (2022).

19. Y. Lin, Q. Hu, M. J. Walter, J. Yang, Y. Meng, X. Feng, Y. Zhuang, R. E. Cohen, H.-K. Mao, Hydrous SiO_2 in subducted oceanic crust and H_2O transport to the core–mantle boundary. *Earth Planet. Sci. Lett.* **594**, 117708 (2022).

20. Y. Tsutsumi, N. Sakamoto, K. Hirose, S. Tagawa, K. Umemoto, Y. Ohishi, H. Yurimoto, Retention of water in subducted slabs under core–mantle boundary conditions. *Nat. Geosci.* **17**, 697–704 (2024).

21. J. Tsuchiya, T. Tsuchiya, S. Tsuneyuki, T. Yamanaka, First principles calculation of a high-pressure hydrous phase, δ-AlOOH. *Geophys. Res. Lett.* **29**, 1909 (2002).

22. W. R. Panero, L. P. Stixrude, Hydrogen incorporation in stishovite at high pressure and symmetric hydrogen bonding in δ-AlOOH. *Earth Planet. Sci. Lett.* **221**, 421–431 (2004).

23. T. Meier, F. Trybel, S. Khandarkhaeva, D. Laniel, T. Ishii, A. Aslandukova, N. Dubrovinskaia, L. Dubrovinsky, Structural independence of hydrogen-bond symmetrisation dynamics at extreme pressure conditions. *Nat. Commun.* **13**, 3042 (2022).

24. M. Millot, F. Coppari, J. R. Rygg, A. Correa Barrios, S. Hamel, D. C. Swift, J. H. Eggert, Nanosecond X-ray diffraction of shock-compressed superionic water ice. *Nature* **569**, 251–255 (2019).

25. V. B. Prakapenka, N. Holtgrewe, S. S. Lobanov, A. F. Goncharov, Structure and properties of two superionic ice phases. *Nat. Phys.* **17**, 1233–1238 (2021).

26. G. Weck, J. A. Queyroux, S. Ninet, F. Datchi, M. Mezouar, P. Loubeyre, Evidence and stability field of fcc superionic water ice using static compression. *Phys. Rev. Lett.* **128**, 165701 (2022).

27. R. J. Husband, H. P. Liermann, J. D. McHardy, R. S. McWilliams, A. F. Goncharov, V. B. Prakapenka, E. Edmund, S. Chariton, Z. Konôpková, C. Strohm, C. Sanchez-Valle, M. Frost, L. Andriambariarijaona, K. Appel, C. Baehtz, O. B. Ball, R. Briggs, J. Buchen, V. Cerantola, J. Choi, A. L. Coleman, H. Cynn, A. Dwivedi, H. Graafsma, H. Hwang, E. Koemets, T. Laurus, Y. Lee, X. Li, H. Marquardt, A. Mondal, M. Nakatsutsumi, S. Ninet, E. Pace, C. Pepin, C. Prescher, S. Stern, J. Sztuk-Dambietz, U. Zastrau, M. I. McMahon, Phase transition kinetics of superionic H_2O ice phases revealed by megahertz X-ray freeelectron laser-heating experiments. *Nat. Commun.* **15**, 8256 (2024).

28. B. Cheng, M. Bethkenhagen, C. J. Pickard, S. Hamel, Phase behaviours of superionic water at planetary conditions. *Nat. Phys.* **17**, 1228–1232 (2021).

29. K. Umemoto, K. Kawamura, K. Hirose, R. M. Wentzcovitch, Post-stishovite transition in hydrous aluminous SiO_2. *Phys. Earth Planet. Inter.* **255**, 18–26 (2016).

30. M. Hou, Y. He, B. G. Jang, S. Sun, Y. Zhuang, L. Deng, R. Tang, J. Chen, F. Ke, Y. Meng, V. B. Prakapenka, B. Chen, J. H. Shim, J. Liu, D. Young Kim, Q. Hu, C. J. Pickard, R. J. Needs, H.-K. Mao, Superionic iron oxide–hydroxide in Earth's deep mantle. *Nat. Geosci.* **14**, 174–178 (2021).

31. J. Li, Y. Lin, T. Meier, Z. Liu, W. Yang, H.-K. Mao, S. Zhu, Q. Hu, Silica-water superstructure and one-dimensional superionic conduit in Earth's mantle. *Sci. Adv.* **9**, eadh3784 (2023).

32. C. Luo, Y. Sun, R. M. Wentzcovitch, Probing the state of hydrogen in δ-AlOOH at mantle conditions with machine learning potential. *Phys. Rev. Res.* **6**, 013292 (2024).

33. Y. He, S. Sun, D. Y. Kim, B. G. Jang, H. Li, H. K. Mao, Superionic iron alloys and their seismic velocities in Earth's inner core. *Nature* **602**, 258–262 (2022).

34. S. C. Zhu, Q. Hu, W. L. Mao, H.-K. Mao, H. Sheng, Hydrogen-bond symmetrization breakdown and dehydrogenation mechanism of FeO_2H at high pressure. *J. Am. Chem. Soc.* **139**, 12139–12132 (2017).

35. R. Tang, J. Liu, D. Y. Kim, H. K. Mao, Q. Hu, B. Yang, Y. Li, C. J. Pickard, R. J. Needs, Y. He, H. Liu, V. B. Prakapenka, Y. Meng, J. Yan, Chemistry and P-V-T equation of state of FeO_2H_x at the base of Earth's lower mantle and their geophysical implications. *Sci. Bullet.* **66**, 1954–1958 (2021).

36. L. Yuan, E. Ohtani, D. Ikuta, S. Kamada, J. Tsuchiya, N. Hirao, Y. Ohishi, A. Suzuki, Chemical reactions between Fe and H_2O up to megabar pressures and implications for water storage in the Earth's mantle and core. *Geophys. Res. Lett.* **45**, 1330–1338 (2018).

37. H. K. Mao, Q. Hu, L. Yang, J. Liu, D. Y. Kim, Y. Meng, L. Zhang, V. B. Prakapenka, W. Yang, W. L. Mao, When water meets iron at Earth's core–mantle boundary. *Natl. Sci. Rev.* **4**, 870–878 (2017).

38. Y. He, D. Y. Kim, V. V. Struzhkin, Z. M. Geballe, V. Prakapenka, H.-K. Mao, The stability of FeH_x and hydrogen transport at Earth's core mantle boundary. *Sci. Bullet.* **68**, 1567–1573 (2023).

39. L. J. Hallis, G. R. Huss, K. Nagashima, G. J. Taylor, S. A. Halldórsson, D. R. Hilton, M. J. Mottl, K. J. Meech, Evidence for primordial water in Earth's deep mantle. *Science* **350**, 795–797 (2015).

40. M. W. Loewen, D. W. Graham, I. N. Bindeman, J. E. Lupton, M. O. Garcia, Hydrogen isotopes in high 3He/4He submarine basalts: Primordial vs. recycled water and the veil of mantle enrichment. *Earth Planet. Sci. Lett.* **508**, 62–73 (2019).

41. V. Fidalgo Cândido, F. Matusalem, M. de Koning, Melting conditions and entropies of superionic water ice: Free-energy calculations based on hybrid solid/liquid reference systems. *J. Chem. Phys.* **158**, 064502 (2023).

42. A. Forestier, G. Weck, F. Datchi, S. Ninet, G. Garbarino, M. Mezouar, P. Loubeyre, X-ray signature of the superionic transition in warm dense fcc water Ice. *Phys. Rev. Lett.* **134**, 076102 (2022).

43. J. A. Queyroux, J. A. Hernandez, G. Weck, S. Ninet, T. Plisson, S. Klotz, G. Garbarino, N. Guignot, M. Mezouar, M. Hanfland, J. P. Itié, F. Datchi, Melting curve and isostructural solid transition in superionic ice. *Phys. Rev. Lett.* **125**, 195501 (2020).

44. T. Kimura, M. Murakami, Revisiting the melting curve of H_2O by Brillouin spectroscopy to 54 GPa. *J. Chem. Phys.* **158**, 134504 (2023).

45. L. Zhang, H. Wang, R. Car, W. E, Phase diagram of a deep potential water model. *Phys. Rev. Lett.* **126**, 236001 (2021).

46. J. M. Brown, T. J. Shankland, Thermodynamic parameters in the Earth as determined from seismic profiles. *Geophys. J. R.y. Astr. Soc.* **66**, 579–96 (1981).

47. T. Komabayashi, S. Omori, S. Maruyama, Petrogenetic grid in the system MgO-SiO_2-H_2O up to 30 GPa, 1600 °C: Applications to hydrous peridotite subducting into the Earth's deep. *J. Geophys. Res.* **109**, B03206 (2004).

48. H. Yuan, L. Zhang, E. Ohtani, Y. Meng, E. Greenberg, V. B. Prakapenka, Stability of Fe-bearing hydrous phases and element partitioning in the system MgO–Al_2O_3–Fe_2O_3–SiO_2–H_2O in Earth's lowermost mantle. *Earth Planet. Sci. Lett.* **524**, 115714 (2019).

49. S. Hull, Superionics: Crystal structures and conduction processes. *Rep. Prog. Phys.* **67**, 1233–1314 (2004).

50. G. W. Brindley, R. Hayami, Kinetics and mechanism of dehydration and recrystallization of serpentine. *Clays Clay Miner.*, **12**, 35–54 (1964).

51. G. C. Maiti, F. Freund, Dehydration-related proton conductivity in kaolinite. *Clay Minerals.* **16**, 395–413 (1981).

52. M. R. Frank, C. E. Runge, H. P. Scott, S. J. Maglio, J. Olson, V. B. Prakapenka, G. Shen, Experimental study of the NaCl-H_2O system up to 28 GPa: Implications for ice-rich planetary bodies. *Phys. Earth Planet. Inter.* **155**, 152–162 (2006).

53. J. A. Hernandez, R. Caracas, S. Labrosse, Stability of high-temperature salty ice suggests electrolyte permeability in water-rich exoplanet icy mantles. *Nat. Commun.* **13**, 3303 (2022).

54. H. Terasaki, E. Ohtani, T. Sakai, S. Kamada, H. Asanuma, Y. Shibazaki, N. Hirao, N. Sata, Y. Ohishi, T. Sakamaki, A. Suzuki, K. Funakoshi, Stability of Fe-Ni hydride after the reaction between Fe-Ni alloy and hydrous phase (δ-AlOOH) up to 1.2Mbar: Possibility of H contribution to the core density deficit. *Phys. Earth Planet Inter.* **194–195**, 18–24 (2012).

55. A. A. P. Koppers, T. W. Becker, M. G. Jackson, K. Konrad, R. D. Müller, B. Romanowicz, B. Steinberger, J. M. Whittaker, Mantle plumes and their role in Earth processes. *Nat. Rev. Earth Environ.* **2**, 382–401 (2021).

56. P. Olson, Z. D. Sharp, Hydrogen and helium ingassing during terrestrial planet accretion. *Earth Planet. Sci. Lett.* **498**, 418–426 (2018).

57. H. Liu, L. Liu, C. Xin, L. Yang, X. Gu, A first-principles study of the structural, electronic and elastic properties of the FeO_2–FeO_2He system under high pressure. *Phys. Chem. Chem. Phys.* **25**, 20225 (2023).

58. S. Ding, P. Zhang, K. Yang, C. Liu, J. Hao, W. Cui, J. Shi, Y. Li, Formation of solid SiO_2 He compound at high pressure and high temperature. *Phys. Rev. B* **106**, 024102 (2022).

59. E. J. Garnero, A. K. McNamara, S. H. Shim, Continent-sized anomalous zones with low seismic velocity at the base of Earth's mantle. *Nat. Geosci.* **9**, 481–489 (2016).

60. S. Ni, E. Tan, M. Gurnis, D. Helmberger, Sharp sides to the African superplume. *Science* **296**, 1850–1852 (2002).

61. S. Labrosse, J. W. Hernlund, N. Coltice, A crystallizing dense magma ocean at the base of the Earth's mantle. *Nature* **450**, 866–869 (2007).

62. E. Tan, M. Gurnis, Metastable superplumes and mantle compressibility. *Geophy. Res. Lett.* **32**, L20307 (2005).

63. I. Mashino, M. Murakami, E. Ohtani, Sound velocities of δ-AlOOH up to core-mantle boundary pressures with implications for the seismic anomalies in the deep mantle. *J. Geophys. Res. Solid Earth* **121**, 595–609 (2016).

64. J. Jiang, F. Zhang, Theoretical studies on the hydrous lower mantle and D″ layer minerals. *Earth Planet. Sci. Lett.* **525**, 115753 (2019).

65. W. Kohn, L. J. Sham, Self-consistent equations including exchange and correlation effects. *Phys. Rev.* **140**, A1133–A1138 (1965).

66. G. Kresse, Efficient iterative schemes for ab initio total-energy calculations using a plane-wave basis set. *Phys. Rev. B* **54**, 11169–11186 (1996).

67. P. E. Blöchl, Projector augmented-wave method. *Phys. Rev. B* **50**, 17953–17979 (1994).

68. J. P. Perdew, K. Burke, M. Ernzerhof, Generalized gradient approximation made simple. *Phys. Rev. Lett.* **77**, 3865–3868 (1996).

69. D. Zhang, H. Bi, F. Z. Dai, W. Jiang, X. Liu, L. Zhang, H. Wang, Pretraining of attention-based deep learning potential model for molecular simulation. *npj Comput. Mater.* **10**, 94 (2024).

70. H. Wang, L. Zhang, J. Han, E. Weinan, DeePMD-kit: A deep learning package for many-body potential energy representation and molecular dynamics. *Comput. Phys. Commun.* **228**, 178–184 (2018).

71. L. Zhang, J. Han, H. Wang, R. Car, E. Weinan, Deep potential molecular dynamics: A scalable model with the accuracy of quantum mechanics. *Phys. Rev. Lett.* **120**, 143001 (2018).

72. J. Zeng, D. Zhang, D. Lu, P. Mo, Z. Li, Y. Chen, M. Rynik, L. Huang, Z. Li, S. Shi, Y. Wang, H. Ye, P. Tuo, J. Yang, Y. Ding, Y. Li, D. Tisi, Q. Zeng, H. Bao, Y. Xia, J. Huang, K. Muraoka, Y. Wang, J. Chang, F. Yuan, S. L. Bore, C. Cai, Y. Lin, B. Wang, J. Xu, J. X. Zhu, C. Luo, Y. Zhang, R. E. A. Goodall, W. Liang, A. K. Singh, S. Yao, J. Zhang, R. Wentzcovitch, J. Han, J. Liu, W. Jia, D. M. York, E. Weinan, R. Car, L. Zhang, H. Wang, DeePMD-kit v2: A software package for deep potential models. *J. Chem. Phys.* **159**,054801 (2023).

73. Y. Zhang, H. Wang, W. Chen, J. Zeng, L. Zhang, H. Wang, DP-GEN: A concurrent learning platform for the generation of reliable deep learning based potential energy models. *Comput. Phys. Commun.* **253**, 107206 (2020).

74. M. Chen, G. C. Guo, L. He, Systematically improvable optimized atomic basis sets for ab initio calculations. *J. Phys. Condens. Matter* **22**, 445501 (2010).

75. P. Li, X. Liu, M. Chen, P. Lin, X. Ren, L. Lin, C. Yang, L. He, Large-scale ab initio simulations based on systematically improvable atomic basis. *Comput. Mater. Sci.* **112**, 503–517 (2016).

76. D. R. Hamann, Optimized norm-conserving Vanderbilt pseudopotentials. *Phys. Rev. B* **88**, 085117 (2013).

77. S. Grimme, J. Antony, S. Ehrlich, H. Krieg, A consistent and accurate ab initio parametrization of density functional dispersion correction (DFT-D) for the 94 elements H-Pu. *J. Chem. Phys.* **132**, 154104 (2010).

78. S. Plimpton, Fast parallel algorithms for short-range molecular dynamics. *J. Comput. Phys.* **117**, 1–19 (1995).

79. T. Schneider, E. Stoll, Molecular-dynamics study of a three-dimensional one-component model for distortive phase transitions. *Phys. Rev. B* **17**, 1302–1322 (1978).

80. W. G. Hoover, Canonical dynamics: Equilibrium phase-space distributions. *Phys. Rev. A* **31**, 1695–1697 (1985).

81. R. Freitas, M. Asta, M. de Koning, Nonequilibrium free-energy calculation of solids using LAMMPS. *Comput. Mater. Sci.* **112**, 333–341 (2016).

82. R. L. Paula, M. de Koning, Nonequilibrium free-energy calculations of fluids using LAMMPS. *Comput. Mater. Sci.* **159**, 316–326 (2019).

83. S. Cajahuaringa, A. Antonelli, Non-equilibrium free-energy calculation of phase-boundaries using LAMMPS. *Comput. Mater. Sci.* **207**, 111275 (2022).

84. X. He, Y. Zhu, A. Epstein, Y. Mo, Statistical variances of diffusional properties from ab initio molecular dynamics simulations. *Npj Comput. Mater.* **4**, 18 (2018).

85. J. Huang, L. Zhang, H. Wang, J. Zhao, J. Cheng, E. Weinan, Deep potential generation scheme and simulation protocol for the $Li_{10}GeP_2S_{12}$-type superionic conductors. *J. Chem. Phys.* **154**, 094703 (2021).

86. J. Sun, B. K. Clark, S. Torquato, R. Car, The phase diagram of high-pressure superionic ice. *Nat. Commun.* **6**, 8156 (2015).

87. C. Liu, H. Gao, Y. Wang, R. J. Needs, C. J. Pickard, J. Sun, H. T. Wang, D. Xing, Multiple superionic states in helium–water compounds. *Nat. Phys.* **15**, 1065–1070 (2019).

88. F. Matusalem, J. S. Rego, M. de Koning, Plastic deformation of superionic water ices. *Proc. Natl. Acad. Sci. U.S.A.* **119**, e2203397119 (2022).

89. K. de Villa, F. González-Cataldo, B. Militzer, Double superionicity in icy compounds at planetary interior conditions. *Nat. Commun.* **14**, 7580 (2023).

90. F. Wu, Y. Sun, T. Wan, S. Wu, R. M. Wentzcovitch, Deep-learning-based prediction of the tetragonal → cubic transition in davemaoite. *Geophys. Res. Lett.* **51**, 12 (2024).

91. S. Menon, Y. Lysogorskiy, J. Rogal, R. Drautz, Automated free-energy calculation from atomistic simulations. *Phys. Rev. Mater.* **5**, 103801 (2021).

92. S. A. Etesami, M. I. Baskes, M. Laradji, E. Asadi, Thermodynamics of solid Sn and PbSn liquid mixtures using molecular dynamics simulations. *Acta Mater.* **161**, 320–330 (2018).
